# Growth-coupled selection of 5-aminolevulinic acid synthetase mutants for 5-aminolevulinic acid biosynthesis in *Corynebacterium glutamicum*

**DOI:** 10.3389/fmicb.2025.1717625

**Published:** 2025-11-18

**Authors:** Peng Yang, Yutian Fan, Yajuan Zhao, Yan Yu, Xinping Yan, Lu Han, Xinqiang Song, An-dong Gong

**Affiliations:** 1 Dabie Mountain Laboratory, College of Tea and Food Science, Xinyang Normal University, Xinyang, Henan, China; 2 Henan Key Laboratory of Tea Plant Biology, College of Tea and Food Science, Xinyang Normal University, Xinyang, Henan, China; 3 Henan International Joint Laboratory of Tea-Oil Tree Biology and High-Value Utilization, College of Tea and Food Science, Xinyang Normal University, Xinyang, Henan, China; 4 College of Life Science, Xinyang Normal University, Xinyang, Henan, China; 5 College of Medicine, Xinyang Normal University, Xinyang, Henan, China

**Keywords:** 5-aminolevulinic acid, 5-aminolevulinic acid synthetase, growth-coupled selection, fermentation optimization, biosynthesis

## Abstract

**Introduction:**

5-Aminolevulinic acid (5-ALA) is a nonprotein amino acid with broad applications in agriculture, medicine, and the food industry. In recent years, substantial efforts have been devoted to enhancing its biosynthesis. The catalytic activity of 5-aminolevulinic acid synthase (ALAS) is a critical determinant of production efficiency.

**Methods:**

In this study, a growth-coupled selection strategy was developed to improve ALAS from *Rhodobacter capsulatus* SB1003 using random and site-specific mutagenesis. The derived ALAS mutant was then introduced into the GRAS-certified producer *Corynebacterium glutamicum*, followed by fermentation optimization.

**Results:**

The enzymatic activity of the best mutant, D4,7,18, increased by 67.41%, leading to 1.18-fold higher 5-ALA accumulation than that of the wild type. Enzymatic analysis suggested that the enhanced activity of D4,7,18 was partly attributable to stronger PLP binding and a lower *K*_m_ for glycine. During fermentation optimization, the results underscored the crucial roles of dissolved oxygen, Fe^2+^ and glycine supplementation. Ultimately, 8.72 g/L of 5-ALA was produced within 60 h, with minimal accumulation of organic acid byproducts.

**Conclusion:**

The growth-coupled selection strategy demonstrated in this study offers a promising approach for optimizing other enzymes and metabolic pathways, provided that an appropriate selection strain and screening conditions are employed.

## 1 Introduction

As the common precursor for the biosynthesis of tetrapyrrole compounds, 5-aminolevulinic acid (5-ALA) is attracting increasing attention due to its wide applications in medicine, agriculture, and cosmetics (Jiang et al., 2022). Several strategies exist for producing this compound. Compared to natural extraction and chemical synthesis, microbial fermentation offers advantages such as simplicity, relatively high yield, and environmental friendliness.

**Figure 1:**
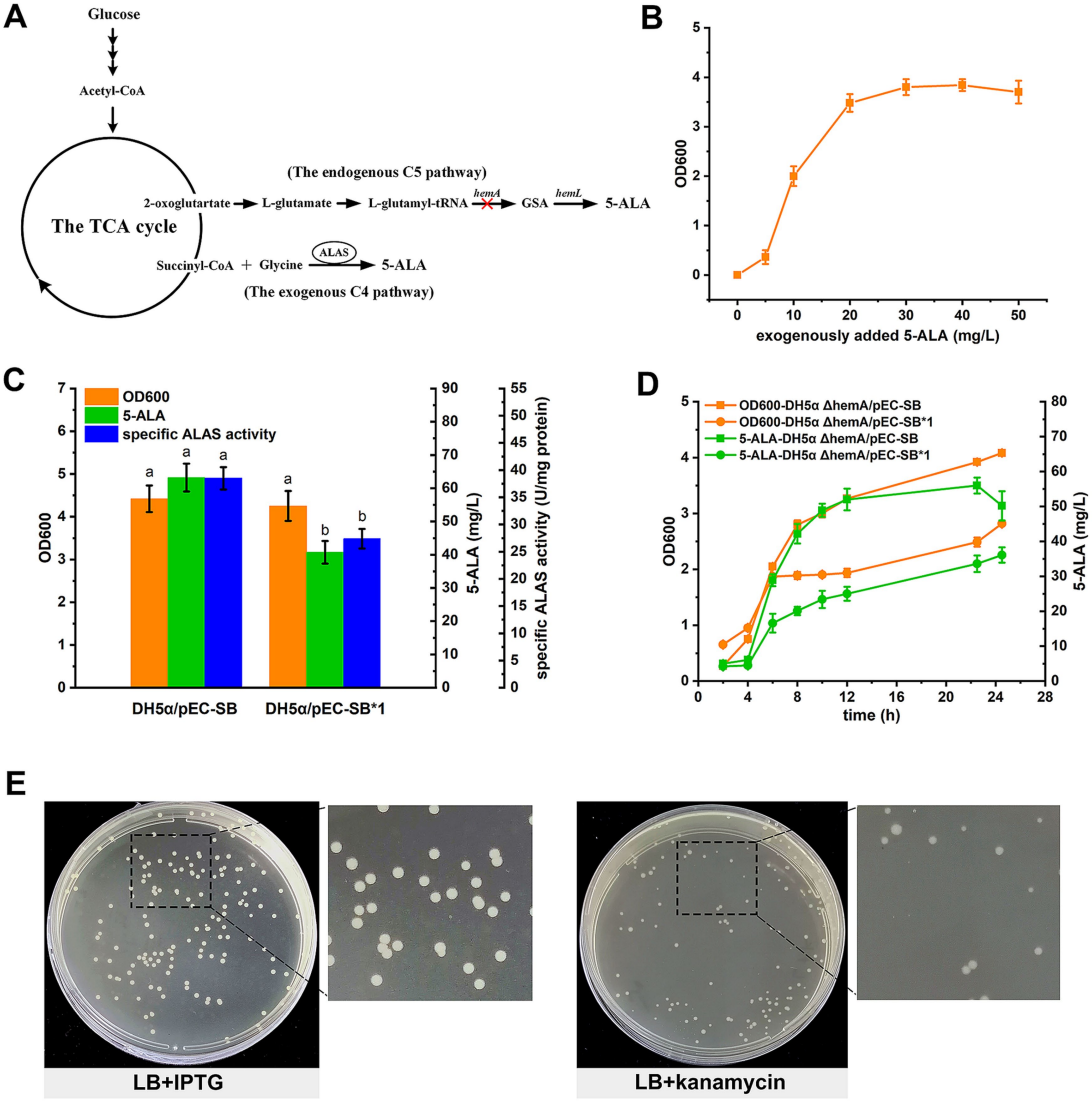
Development of a growth-coupled screening strategy for ALAS mutants. **(A)** The 5-ALA biosynthetic pathway in *E. coli*. The *hemA* gene of the C5 route was deleted to construct a selection strain. **(B)** Relationship between the growth of the 5-ALA auxotrophic strain and exogenously added 5-ALA. **(C)** Comparison of the two ALASs in *E. coli* DH5α. Different letters above the bars indicate significant differences (*p* < 0.05). **(D)** Comparison of the two ALASs in DH5α ΔhemA. **(E)** LB containing kanamycin was more suitable for mutant screening. In **(B–D)**, results are averages from three independent experiments, with standard deviations indicated by error bars.

In living organisms, two types of metabolic pathways are involved in ALA synthesis: the tightly regulated C5 pathway and the relatively straightforward C4 pathway (Figure 1A). The C5 pathway occurs in algae, higher plants, and many bacteria, including *Escherichia coli*. In this pathway, L-glutamate is first transformed into L-glutamyl-tRNA by glutamyl-tRNA synthetase, which is then used to synthesize 5-ALA through the actions of glutamyl-tRNA reductase (encoded by *hemA*) and glutamate-1-semialdehyde aminotransferase (encoded by *hemL*). In contrast, the C4 pathway, found in birds, mammals, yeast, and purple non-sulfur photosynthetic bacteria, utilizes 5-aminolevulinic acid synthetase (ALAS) to directly condense glycine and succinyl-CoA, forming 5-ALA.

The enzymes of the C5 pathway are tightly regulated at multiple levels, including transcriptional, post-transcriptional, and translational levels, making efficient 5-ALA biosynthesis challenging (Dailey et al., 2017). Efforts have been made to improve 5-ALA production by regulating metabolic balance, weakening downstream pathways, screening key enzymes, and enhancing the stability of the producer strain (Zhang et al., 2019; Aiguo and Meizhi, 2019; Cui et al., 2019; Luo et al., 2022; Zhang et al., 2020).

On the other hand, 5-ALA production through the C4 pathway has gained more attention due to its simplicity. In the C4 pathway, ALAS plays a critical role in determining the 5-ALA titer. Screening this enzyme from diverse sources has proven to be an effective strategy for enhancing 5-ALA biosynthesis (Zhang et al., 2013; Yang et al., 2016; Chen et al., 2020). Alternatively, selecting ALAS mutants with increased activity offers another promising approach. Lendrihas et al. mutated an ALAS from *Rhodobacter capsulatus* by targeting the active site loop sequence (Lendrihas et al., 2010). Using a 5-ALA-deficient *E. coli* strain, they obtained ALAS mutants with improved porphyrin production and enzyme activity, although their capacity for 5-ALA production was not determined.

In another study, the thermostability of an *R. palustris*-sourced ALAS was improved by replacing histidines with stabilizing residues, which also reduced hemin inhibition (Tan et al., 2019). Two mutants, H29R and H15K, with slightly increased 5-ALA synthesis capacity were derived through computer-aided rational design. Ikushiro et al. engineered an ALAS from *Caulobacter crescentus* by substituting the heme-binding site residues H340 or C398 with glycine. The mutants exhibited resistance to heme inhibition, which may have potential for enhancing 5-ALA production (Ikushiro et al., 2018). In another study, cysteine-targeted mutations were used to overcome heme inhibition (He et al., 2022). The resulting mutants, RP-C132A and RC-C201A, exhibited increased enzyme activity and reduced heme inhibition. 5-ALA production using these mutants was improved by 14.0 and 21.6%, respectively. Recently, Wang et al. designed a biosensor that utilizes the reduction in cAMP levels resulting from 5-ALA accumulation (Wang Q. et al., 2024). ALAS and *E. coli* strain mutants were obtained through this biosensor-facilitated screening, and combined with traditional metabolic engineering strategies, the 5-ALA titer reached 58.54 g/L in a 5-L fermenter. Recent advancements in metabolic engineering for 5-ALA production via the C4 pathway are shown in Table 1.

**Table 1:** Research progress in 5-ALA biosynthesis via the C4 pathway in engineered microorganisms.

Strain and strategy^a^	Substrates	Titer (g/L)	Productivity (g/L/h)	References
*E. coli*				
Overexpression of *hemA^Rs^*	Succinate, glycine	5.2^b^	0.43	Xie et al. (2003)
Overexpression of *hemA^Ar^*	Glucose, succinate, glycine, xylose	7.3^b^	0.24	Lin et al. (2009)
Inhibition of 5-ALA dehydratase by glucose	Glucose, succinate, glycine	3.1^b^	0.11	Liu et al. (2010)
Overexpression of *hemA^Ar^*, short-term dissolved oxygen shock	Glucose, succinate, glycine	9.4^b^	0.43	Jun et al. (2013)
Overexpression of *hemA^Rp^*, CAT and SOD	Glucose, succinate, glycine	11.5^b^	0.52	Zhu et al. (2019)
Biosensor facilitated *hemA* mutation and strain evolution, metabolic pathway engineering	Glucose, glycine	58.54^b^	1.58	Wang Q. et al. (2024)
Overexpression of *hemA^Kd^*, model-guided systematic engineering, fine-tuning redox homeostasis, fermentation optimization	Glucose, glycine	63.39^b^	1.44	Zhou et al. (2024)
*C. glutamicum*				
Overexpression of *hemA^Rs^*, *ppc* and *rhtA*, blocking by-products pathways, deletion of highmolecular-weight penicillin-binding proteins	Glucose, glycine	7.5^b^	0.23	Feng et al. (2016)
Overexpression of *hemA^Rs^* and the glycine synthesis pathway (*serABC*, *glyA*), inactivation of L-serine dehydratase	Glucose, glycine	3.4^c^	0.28	Zou et al. (2017)
Moderate overexpression of *hemA^Rp^* and *ppc*	Glucose, glycine	16.3^b^	0.42	Chen et al. (2020)
Overexpression of *hemA^Rc^*, inactivation of glutamate dehydrogenase and isocitrate dehydrogenase, fermentation optimization	Glucose, glycine	5.6^c^	0.12	Ge et al. (2021)
Increase succinyl-CoA supply, fusion overexpression of SucCD and a heme feedback-resistent HemA*^Rp^*, 8% dissolved oxygen	Glucose, glycine	3.8^b^	0.05	Zhao et al. (2025)
Overexpression of mutant *hemA^Rc^*, deletion of *sucCD*, fermentation optimization	Glucose, glycine	8.72^c^	0.15	This study

a hemA^Rs^, hemA from Rhodobacter sphaeroide; hemA^Ar^, hemA from *Agrobacterium radiobacter*; hemA^Rp^, hemA from *Rhodopseudomonas palustris*; hemA^Kd^, hemA from Komagataeibacter diospyri; hemA^Rc^, hemA from *Rhodobacter capsulatus*.

b Titer obtained from bioreactor fermentation.

c Titer obtained from shake-flask fermentation.

To acquire the target mutants, an efficient screening method is crucial. Coupling metabolite production to cell growth has been a long-standing approach in bioengineering. Typically, a growth-deficient selection strain is first constructed and then rescued by the enzymes, modules, or pathways being evaluated during selection. In this manner, the growth of the selection strain serves as a proxy for the efficiency of the tested elements (Li et al., 2023). This strategy is less device-dependent and is well-suited for products without obvious phenotypic markers, such as color changes or antibiotic resistance.

Our previous study demonstrated that the codon-optimized ALAS from *R. capsulatus* SB1003 exhibited significant potential for 5-ALA biosynthesis in *Corynebacterium glutamicum* (Yang et al., 2016). Building on these findings and inspired by the aforementioned studies, we sought to enhance ALAS through mutation and growth-coupled screening in this study.

## 2 Materials and methods

### 2.1 Bacterial strains, plasmids, and media

The strains and plasmids used in this study are shown in Table 2. *E. coli* strains were propagated in Luria-Bertani (LB) medium at 37 °C with aeration. The DH5α ΔhemA strain was constructed via Red recombination using the one-step inactivation method (with primers hemA-F and hemA-R) and grown on LB supplemented with 30 μg/mL 5-ALA (Datsenko and Wanner, 2000). Detailed information on the primers is provided in Supplementary Table S1. A modified minimal medium was used for 5-ALA fermentation, which contained 16 g/L (NH_4_)_2_SO_4_, 3 g/L KH_2_PO_4_, 16 g/L Na_2_HPO_4_·12H_2_O, 1 g/L MgSO_4_·7H_2_O, 0.01 g/L MnSO_4_·7H_2_O and 2 g/L yeast extract. Glucose was added as the carbon source. *C. glutamicum* CgS1 was propagated in BHIS medium (2.5 g/L beef extract, 5 g/L tryptone, 5 g/L NaCl, 18.5 g/L brain heart infusion, 91 g/L sorbitol) at 30 °C with aeration. For 5-ALA fermentation, CGXII medium was used (20 g/L (NH_4_)_2_SO_4_, 5 g/L urea, 1 g/L KH_2_PO_4_, 1 g/L K_2_HPO_4_, 0.25 g/L MgSO_4_·7H_2_O, 42 g/L MOPS (3-morpholinopropanesulfonic acid), 10 mg/L CaCl_2_, 10 mg/L FeSO_4_·7H_2_O, 10 mg/L MnSO_4_·H_2_O, 1 mg/L ZnSO_4_·7H_2_O, 0.2 mg/L CuSO_4_, 0.02 mg/L NiCl_2_·6H_2_O, and 0.03 g/L protocatechuate). Kanamycin (25 μg/mL) and isopropyl β-D-1-thiogalactopyranoside (IPTG, 0.1 mM) were added as needed.

**Table 2:** Strains and plasmids used in this work.

Strains and plasmids	Relevant characteristics	References
Strains		
*E. coli* DH5α	Wild type, subcloning host	Transgen
DH5α ΔhemA	*E. coli* DH5α Δ*hemA*	This study
*E. coli* BL21(DE3)	*hsdS*, *gal* (Δ*cIts857 ind1*, *Sam7*, *nin5*, *lacUV5-T7*, *gene1*)	Transgen
CgS1	*C. glutamicum* ATCC 13032 Δ*sucCD*	Yang et al. (2016)
Plasmids		
pEC-XK99E	Kan^r^; *E. coli*-*C. glutamicum* shuttle vector	Lab stock
pEC-SB	Kan^r^; pEC-XK99E carrying ALAS from *R. capsulatus* SB1003	Yang et al. (2016)
pEC-SB*1	Kan^r^; pEC-XK99E carrying ALAS^H342A^	Lab stock
pET28a	Kan^r^, f1 ori, T7 promoter	Lab stock
pET28a-SB	pET28a carrying the wild type ALAS	This study
pET28a-SB4	pET28a carrying ALAS^E27V^	This study
pET28a-SB5	pET28a carrying ALAS^A227T, H237D^	This study
pET28a-SB7	pET28a carrying ALAS^G105C^	This study
pET28a-SB13	pET28a carrying ALAS^P367A, R368K^	This study
pET28a-SB15	pET28a carrying ALAS^P367K, R368K^	This study
pET28a-SB18	pET28a carrying ALAS^F363Y^	This study
pEC-D4,7	Kan^r^; pEC-XK99E carrying ALAS^E27V, G105C^	This study
pEC-D4,18	Kan^r^; pEC-XK99E carrying ALAS^E27V, F363Y^	This study
pEC-D7,18	Kan^r^; pEC-XK99E carrying ALAS^G105C, F363Y^	This study
pEC-D4,7,18	Kan^r^; pEC-XK99E carrying ALAS^E27V, G105C, F363Y^	This study

### 2.2 Mutation and selection of ALAS

Error-prone PCR was performed using the GeneMorph II Random Mutagenesis kit (Agilent Technologies, United States) following the manufacturer’s instructions. The randomly mutated *hemA* gene was amplified from pEC-SB using the primers sbmF and sbmR, then cloned into pEC-XK99E digested with *Eco*RI and *Bam*HI via the SLIC method (Jeong et al., 2012). The assembly mixture was transformed into DH5α ΔhemA and plated onto LB agar supplemented with kanamycin. After 48 h of cultivation, the largest 10% of colonies were selected and sequenced.

For active site loop mutation, degenerate primers were used. PCR was performed with the primer pairs mSB-F1 and mSB-R1 (producing a 3.4 kb product) or mSB-F2 and mSB-R2 (producing a 4.8 kb product), using pEC-SB as the template. The two fragments were assembled using the SLIC method, transformed into DH5α ΔhemA, and subsequently screened and sequenced.

To combine the mutation sites of plasmids 4, 7 and 18, PCR was performed using the primer combinations of sbmF, c-r, c-r2, c-f, c-f2, mSB-R2, mSB-F3 and sbmR. The fragments containing mutation sites were cloned into *Eco*RI- and *Bam*HI-digested pEC-XK99E using the SLIC method, transformed into DH5α, and subsequently screened and sequenced.

### 2.3 Strain cultivation

For 5-ALA fermentation in *E. coli*, a single colony was inoculated into 5 mL of LB medium supplemented with kanamycin. After 12 h of cultivation at 37 °C and 200 rpm, the preculture was transferred into a test tube containing 5 mL of LB medium with a 1% inoculum. Fermentation was carried out at 37 °C and 250 rpm. For shake flask fermentation, the preculture was inoculated into 50 mL of 5-ALA fermentation medium containing kanamycin, with a 4% inoculum. Fermentation was performed at 37 °C and 250 rpm, and 0.1 mM IPTG, 2 g/L glycine, and 20 g/L glucose were added when the OD600 reached approximately 0.6. Glycine was added at 2 g/L every 12 h, and the pH was maintained at 6.5 with ammonium hydroxide.

For 5-ALA fermentation in *C. glutamicum*, a single colony was inoculated into 4 mL of BHIS medium containing kanamycin and incubated at 180 rpm and 30 °C for 20 h. The preculture was then transferred into a 1-L baffled shake flask containing 50 mL of CGXII medium with 0.01% defoaming agent, 10 μg/L biotin, and 130 g/L glucose. IPTG was added after 6 h. Cultivation was carried out at 30 °C and 180 rpm, with the pH maintained at 6.5 using ammonium hydroxide. The precursor glycine was added at 2 g/L every 12 h.

### 2.4 Measurement of ALAS activity

Mutants were cloned into pET28a and expressed in *E. coli* BL21(DE3) for SDS-PAGE and enzymatic assays. A single colony was inoculated into 5 mL of LB medium containing kanamycin and incubated for 12 h at 37 °C and 200 rpm. The preculture was then transferred to 50 mL of LB medium containing kanamycin and 0.1 mM IPTG, and incubated for 12 h at 30 °C and 250 rpm. Cells were harvested by centrifugation at 6,000 rpm for 8 min at 4 °C, washed in 50 mM Tris–HCl buffer (pH 7.5), and disrupted by sonication. The crude extract was centrifuged at 12,000 rpm for 10 min at 4 °C, and the supernatant was used for enzymatic purification using a Ni-NTA Agarose column (1 cm diameter, 1 mL volume, Bio-Rad and Qiagen). The assay mixture contained 50 μL of 1 M glycine, 50 μL of 2 mM succinyl-CoA, 6 μL of 1 M Tris buffer (pH 7.5), and 14 μL of 10 mM pyridoxal phosphate. One unit of enzyme activity was defined as the amount of enzyme that catalyzed the formation of 1 nmol of 5-ALA per minute at 37 °C. The thermostability of ALAS was evaluated by measuring the residual activities after 120 min incubation at 30 °C. The pH stability of recombinant ALAS was evaluated after incubation at various pH values (pH 6.5–8.5) for 60 min at 4 °C. Protein concentrations were measured using the Bradford method, with bovine serum albumin as the standard (Bradford, 1976).

Kinetic parameters were determined by measuring enzyme activity at different concentrations of succinyl-CoA (5–150 μM) and glycine (1–40 mM). *K*_m_ and *V*_max_ values were calculated by fitting the data to the Michaelis–Menten equation using nonlinear regression (Origin 8, OriginLab, United States). The *k*_cat_ value was calculated using the equation *k*_cat_ = *V*_max_/[E], where [E] is the molar concentration of the enzyme used in the experiment.

### 2.5 Analytical methods

Optical density (OD) was measured at 600 nm with a spectrophotometer (Shimadzu, Japan). Glucose and organic acids were quantitatively assessed by high-performance liquid chromatography (HPLC) (Shimadzu, Japan) with a refractive index detector (RID-10A, Shimadzu, Japan), as described previously (Yang et al., 2016). 5-ALA levels were measured using modified Ehrlich’s reagent (Kang et al., 2011). Glutamate was determined using a bioanalyzer (SBA-40D, Institute of Shandong Academy of Sciences, China). Secreted porphyrin compounds in the cell-free medium were estimated using a spectrophotometer at 405 nm (Soret band) and 495 nm (collective Q-bands).

### 2.6 Statistical analysis

Statistical analyses were performed using SPSS for Windows (version 13.0). A *p* value of <0.05 was considered statistically significant.

## 3 Results

### 3.1 Design of a growth-coupled screening strategy for ALAS mutants

In this study, a 5-ALA auxotrophic *E. coli* strain was constructed by deleting *hemA*^C5^ and used as the selection strain (Figure 1A). *E. coli* synthesizes 5-ALA exclusively via the C5 pathway, and 5-ALA is an essential precursor for porphyrin, heme and vitamin biosynthesis. Given the involvement of glutamyl-tRNA in protein synthesis, glutamyl-tRNA reductase (*hemA* ^C5^) was chosen as the target to minimize disruption of this process. Disruption of the C5 pathway rendered the strain auxotrophic for 5-ALA. As shown, the DH5α ΔhemA strain does not grow on LB medium unless supplemented with exogenous 5-ALA (Figure 1B).

In our design, the growth defect was complemented by heterologous expression of ALAS mutants. Meanwhile, biomass accumulation in the selection strain served as a proxy for ALAS mutant activity. As a proof of concept, both the wild-type ALAS and a mutant were used to test the feasibility of the screening strategy. The H342A mutant (plasmid pEC-SB*1) exhibited reduced activity and lower 5-ALA accumulation, yet showed biomass comparable to the wild type (plasmid pEC-SB) when expressed in *E. coli* DH5α (Figure 1C). However, when expressed in the selection strain, both biomass and 5-ALA accumulation were higher in DH5α ΔhemA/pEC-SB, indicating a coupling between growth and 5-ALA production (Figure 1D).

To mimic a selection process, cultures of DH5α ΔhemA/pEC-SB and DH5α ΔhemA/pEC-SB*1 were mixed in equal proportions (based on biomass) and spread onto LB agar plates containing both IPTG and kanamycin after appropriate dilution. However, few colonies appeared, possibly due to growth pressure (data not shown). Subsequently, LB agar containing only IPTG was tested, but differences between the two types of colonies were minimal (Figure 1E). Finally, LB agar supplemented with kanamycin alone was used, which successfully differentiated the colonies (Figure 1E). The ten largest and ten smallest colonies were picked and subjected to DNA sequencing. All of the large colonies were identified as DH5α ΔhemA/pEC-SB, while 9 out of 10 of the small colonies were DH5α ΔhemA/pEC-SB*1. These results further confirmed the feasibility of the growth-coupled screening strategy.

### 3.2 Improving ALAS through growth-coupled screening

**Figure 2:**
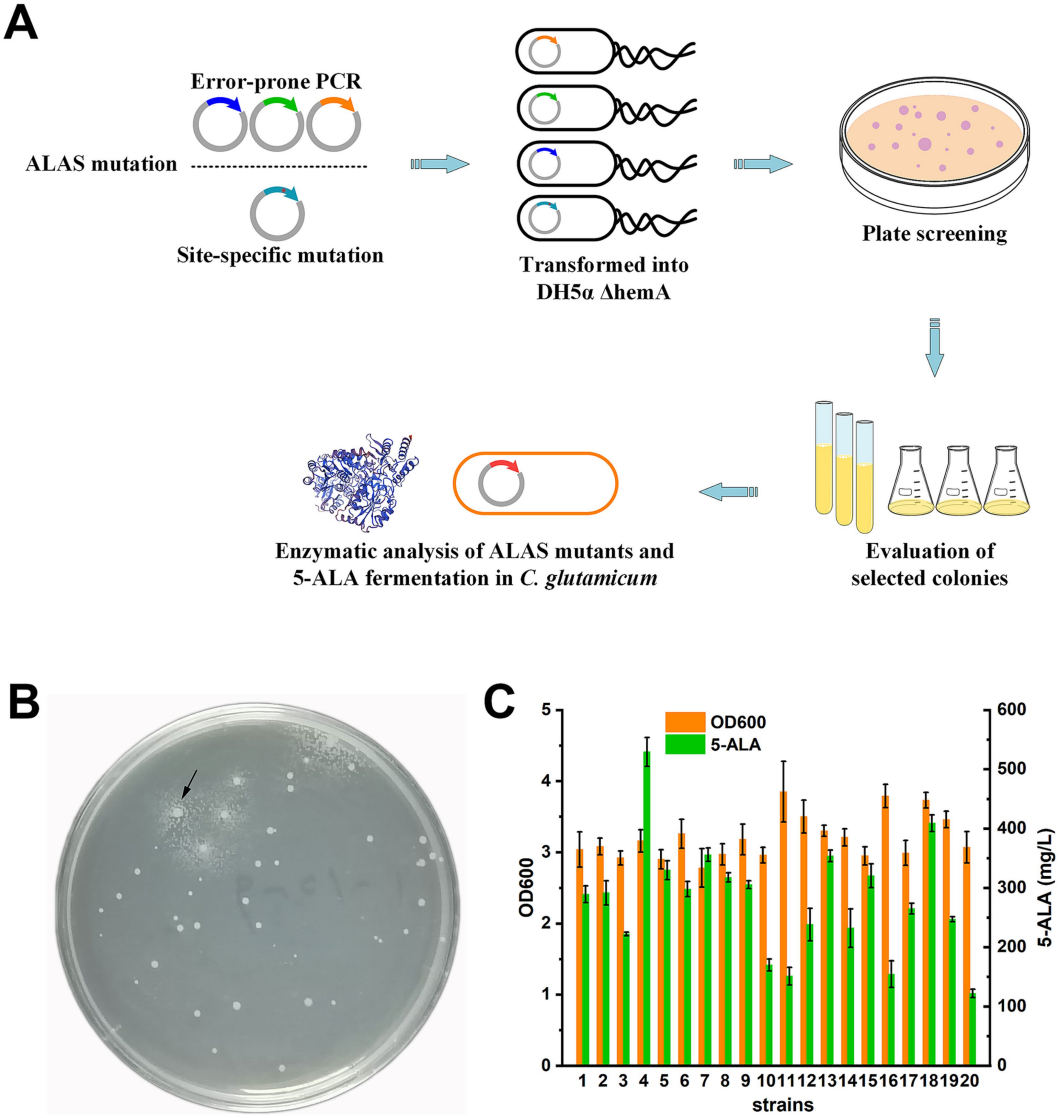
ALAS mutants screening. **(A)** Work flow of the growth-coupled screening. **(B)** A typical screening plate. One potential target mutant was shown by the arrow. **(C)** Evaluation of selected strains carrying ALAS mutants using shake-flask fermentation. Information on the initial strain screening is provided in Supplementary Table S2. Results are averages from three independent experiments, with standard deviations indicated by error bars.

To improve the enzymatic properties of ALAS and enhance 5-ALA biosynthesis, the encoding gene *hemA*^C4^ was mutated via either random mutagenesis or site-directed mutagenesis, followed by screening (Figure 2A). For random mutagenesis, the *hemA*^C4^ gene was subjected to error-prone PCR. For site-directed mutagenesis, the active site loop of ALAS—which plays a major role in catalysis—was targeted by modifying imperfectly conserved residues (Supplementary Figure S1).

After transformation into DH5α ΔhemA, colonies of varying sizes appeared after 48 h of cultivation. On a typical screening plate, small satellite colonies were observed surrounding some larger colonies (Figure 2B). When streaked onto fresh plates, these satellite colonies failed to grow, indicating that 5-ALA secreted by the larger colonies had rescued their growth defect during selection. A total of 200 large colonies (particularly those with satellite colonies) were selected from 60 plates (~1.2 × 10^5^ colonies in total) and evaluated in test-tube cultivation (Supplementary Table S2). Following primary screening, the 20 strains with the highest 5-ALA/OD values were selected for shake-flask fermentation (Supplementary Table S2; Figure 2C). The top-performing mutant (mutant 4) produced 529.18 ± 24.3 mg/L of 5-ALA after 48 h.

**Figure 3:**
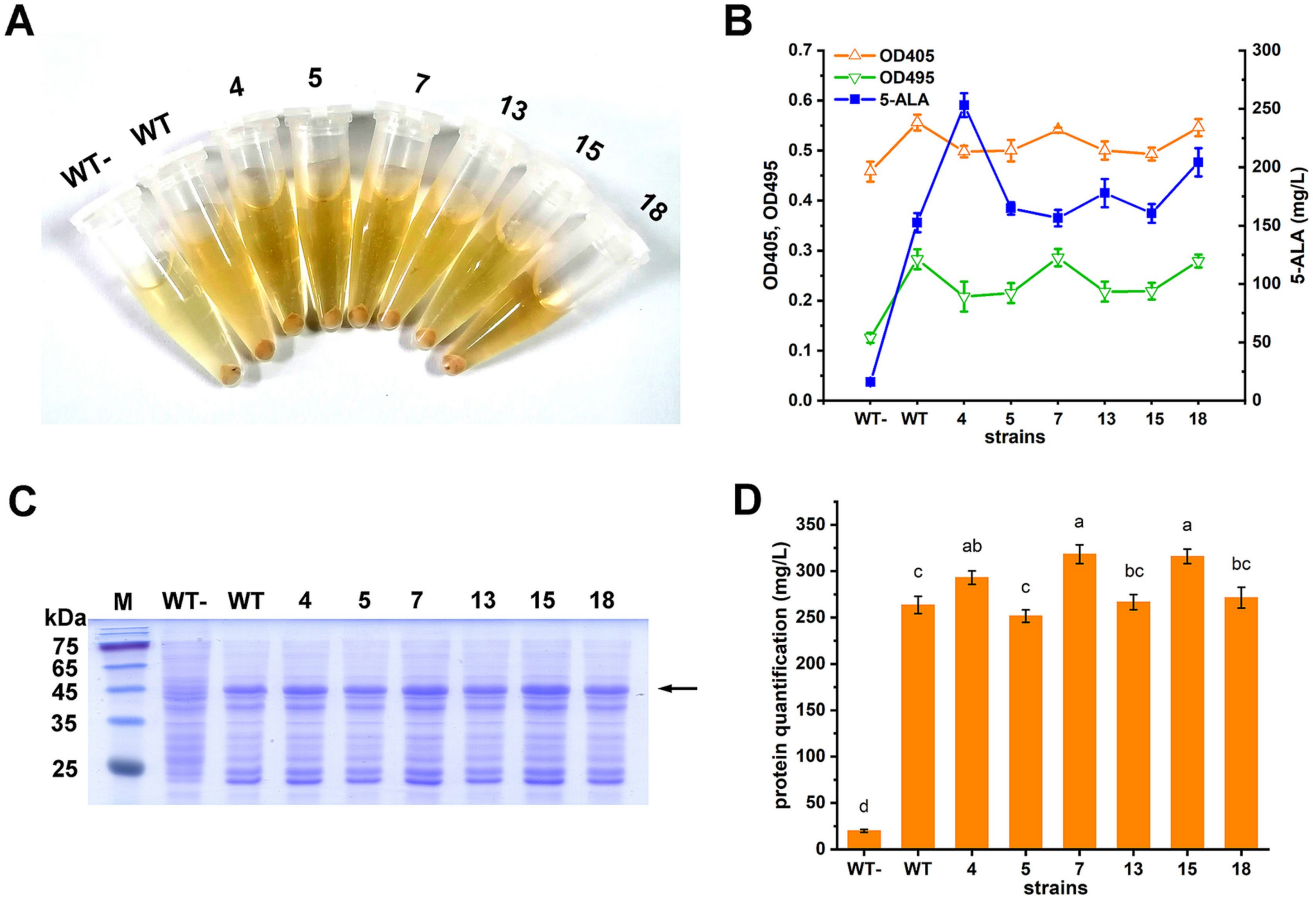
Characterization of the ALAS mutants. **(A)** Cell pellets and supernatants of the strains cultivated in LB. WT-, strain carrying the wild type ALAS without IPTG induction; WT, strain carrying the wild type ALAS; 4, 5, 7, 13, 15, and 18, strains carrying the selected ALAS mutants. **(B)** 5-ALA and porphyrin compounds accumulation. A modified minimal medium was used, as porphyrin detection was interfered with by the LB medium. **(C)** SDS-PAGE of total soluble protein. The ALAS mutants were expressed in *E. coli* BL21(DE3). Lane 1, protein marker; lane 2, strain carrying the wild type ALAS without IPTG induction; lane 3, strain carrying the wild type ALAS; lane 4–9, strains carrying the selected ALAS mutants. The ALAS band was indicated by the arrow. **(D)** Quantitative comparison of proteins in **(C)**. The quantification was performed by Bio-Rad Quantity One Version 4.6.8. Results are averages from three independent experiments, with standard deviations indicated by error bars. Different letters above the bars indicate significant differences (*p* < 0.05).

Six mutants (4, 5, 7, 13, 15, and 18) with the highest 5-ALA/OD values (*p* < 0.05) were further selected and compared with the wild-type ALAS in terms of 5-ALA and downstream porphyrin accumulation. When ALASs were expressed, both the cell pellets and supernatants turned pink or red. As a negative control, the strain harboring the wild-type ALAS without induction showed no color change (Figure 3A). However, the color differences among the ALAS variants were difficult to distinguish by the naked eye. Porphyrin accumulation was assessed by measuring the Soret band (OD_405_) and the collective Q-bands (OD_495_). As shown, OD_405_ and OD_495_ values were generally consistent with each other, but did not always correlate with 5-ALA titers (Figure 3B). Since the culture color was primarily caused by downstream porphyrin derivatives, the results of this study suggest that porphyrins are not suitable as direct selection markers for screening high 5-ALA producers.

The six mutants were sequenced, and their corresponding enzyme activities were determined (Table 3). All mutants exhibited increased 5-ALA accumulation, and five showed enhanced enzymatic activity. Mutant 15, however, displayed slightly reduced but comparable activity relative to the wild type. Protein expression levels of the mutants were also assessed. Mutants 4, 7, and 15 showed moderate increases (*p* < 0.05), while the changes in the other mutants were not statistically significant (Figures 3C,D).

**Table 3:** Characterization of the ALAS mutants.^a^

Variants	Mutation	5-ALA/OD600 (mg/L)	Specific enzyme activity (U/mg protein)
Wild type	–	47.98 ± 3.52	36.51 ± 1.28
4	E27V	90.42 ± 5.36	46.43 ± 2.13
5	A227T, H237D	66.81 ± 2.68	40.21 ± 3.35
7	G105C	82.50 ± 2.73	45.47 ± 2.14
13	P367A, R368K	63.97 ± 5.08	42.33 ± 4.24
15	P367K, R368K	54.41 ± 4.45	34.32 ± 1.98
18	F363Y	75.67 ± 4.13	43.01 ± 2.32
D4,7	E27V, G105C	95.24 ± 3.78	49.17 ± 2.44
D4,18	E27V, F363Y	64.65 ± 3.11	41.26 ± 3.21
D7,18	G105C, F363Y	87.21 ± 4.42	55.83 ± 1.84
D4,7,18	E27V, G105C, F363Y	104.42 ± 3.11	61.12 ± 4.27

a The mutants were cloned into pET28a and expressed in *E. coli* BL21(DE3). The strains were cultivated in 50 mL of LB medium containing kanamycin and 0.1 mM IPTG at 30 °C and 250 rpm for 12 h. The data are presented as the mean ± standard deviation of three independent experiments.

To further improve ALAS, combinatorial mutations of the three top-performing variants (4, 7, and 18) were constructed. As shown in Table 3, the resulting triple mutant D4,7,18 produced 1.18-fold more 5-ALA than the wild type, along with a 67.41% increase in enzymatic activity. The triple mutant exhibited enhanced performance compared to the single and double mutants, indicating a synergistic effect of the three mutation sites.

To illustrate the mechanism underlying the enhanced enzymatic properties of mutant D4,7,18, molecular docking was performed to examine the interactions between ALAS and its substrates. According to calculations with FoldX software, the ΔΔG of mutant D4,7,18 was 0.08 kcal/mol, indicating a neutral effect on the overall stability of ALAS (Delgado et al., 2019). The binding energy between PLP, the essential cofactor in catalysis, and ALAS was determined using AutoDock Vina software (Morris et al., 2009). The ΔΔG of mutant D4,7,18 was −1.43 kcal/mol, suggesting enhanced PLP binding.

**Figure 4:**
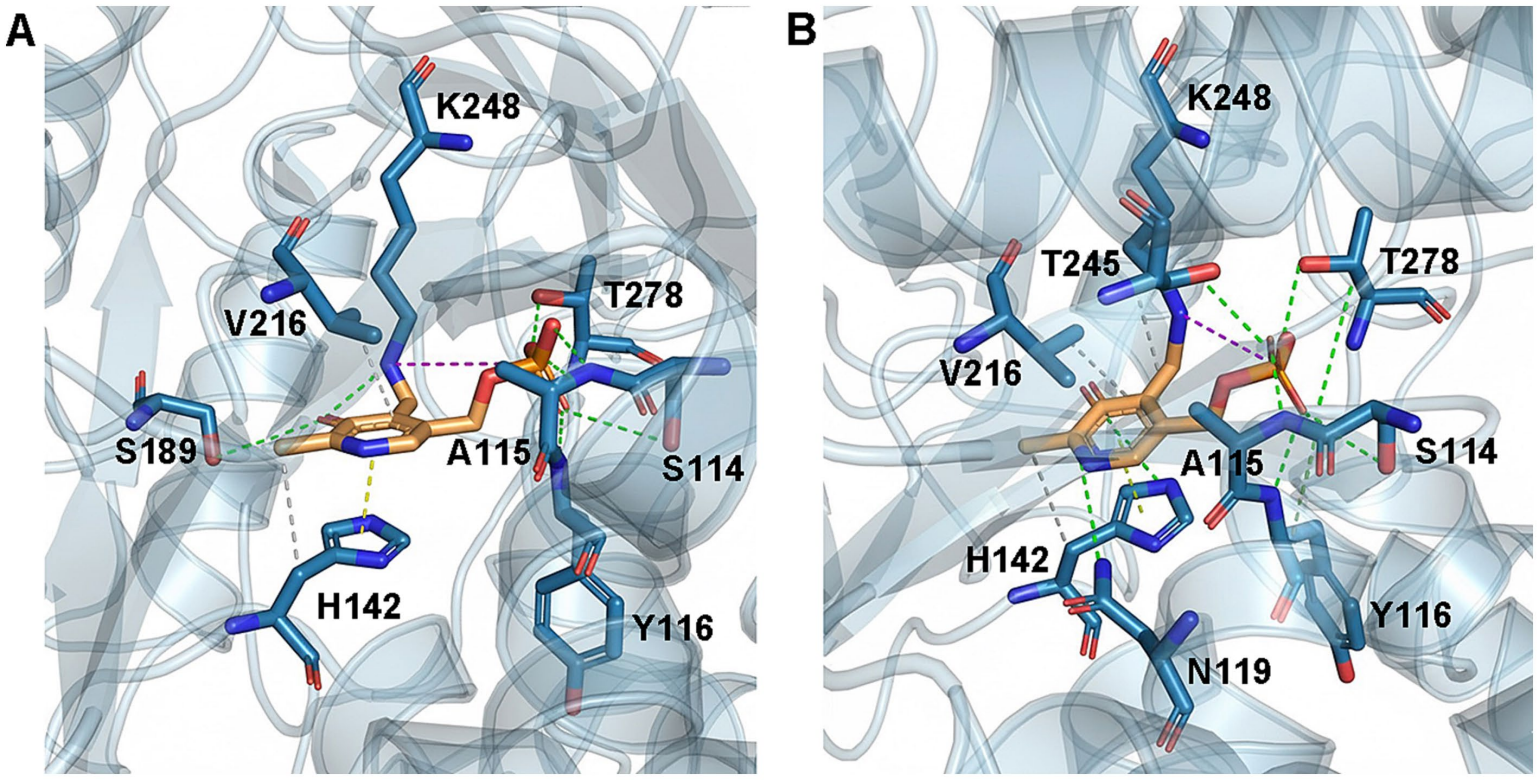
WT and D4,7,18 structural analysis for PLP binding. Hydrophobic interactions (dash lines in gray), hydrogen bonds (dash lines in green), *π*-stacking (dash lines in yellow) and salt bridges (dash lines in pink) were shown in **(A)** WT–PLP complexes and **(B)** D4,7,18–PLP complexes. Molecular docking was performed using AutoDock Vina software and protein-ligand interactions were predicted by PLIP (Schake et al., 2025).

**Figure 5:**
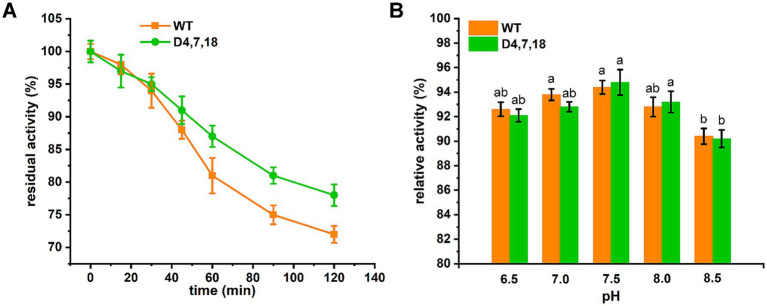
Stability of recombinant ALAS. **(A)** Thermal stability of recombinant ALAS was evaluated by incubating the enzymes at 30 °C for up to 120 min. The activity measured before incubation was defined as 100%. **(B)** pH stability of recombinant ALAS was determined after incubation at various pH values for 60 min at 4 °C. The residual activity was measured under standard assay conditions (pH 7.5, 37 °C), and the activity of the non-treated control was arbitrarily set to 100%. Results are averages from three independent experiments, with standard deviations indicated by error bars. Different letters above the bars indicate significant differences (*p* < 0.05).

In the case of D4,7,18, PLP formed additional hydrogen bonds (with residues N119 and T245) and hydrophobic interactions (with residue K248), which may contribute to the catalysis of ALAS (Figure 4). Furthermore, steady-state kinetics revealed that the *K*_m_(Gly) value of mutant D4,7,18 decreased slightly compared with the wild type (2.13 vs. 2.66 mM), resulting in a 33.68% increase in specificity (*k*_cat_/*K*_m_) for glycine (Table 4). The temperature and pH stabilities of D4,7,18 are shown in Figure 5. After incubation for 120 min at 30 °C, the wild-type ALAS retained approximately 72.21% of its initial activity, whereas the D4,7,18 mutant retained about 78.16%, indicating a slightly improved thermostability (Figure 5A). Both the wild-type and mutant enzymes were stable within the pH range of 6.5 to 8.0. After incubation for 60 min at 4 °C, more than 90% of the relative activity was retained (Figure 5B). However, at pH 8.5, both enzymes were less stable. These results indicate that the D4,7,18 mutant exhibits good stability under the fermentation conditions of 30 °C and pH 6.5. Altogether, an ALAS mutant with enhanced catalytic capacity was identified through growth-coupled screening.

**Table 4:** Kinetic parameters for wild-type and D4,7,18.^a^

ALASs	*k*_cat_(s^−1^)	*K*_m_(Gly) (mM)	*k*_cat_/*K*_m_(Gly) (s^−1^ mM^−1^)	*K*_m_(succinyl-CoA) (μM)	*k*_cat_/*K*_m_ (succinyl-CoA) (s^−1^ μM^−1^)
Wild type	2.52 ± 0.22	2.66 ± 0.26	0.95 ± 0.23	17.5 ± 2.65	0.14 ± 0.03
D4,7,18	2.94 ± 0.15	2.13 ± 0.22	1.27 ± 0.18	16.88 ± 2.21	0.17 ± 0.02

a Kinetic parameters were determined at 37 °C using purified enzyme. The data are presented as the mean ± standard deviation of three independent experiments.

### 3.3 5-ALA biosynthesis in *Corynebacterium glutamicum*

**Figure 6:**
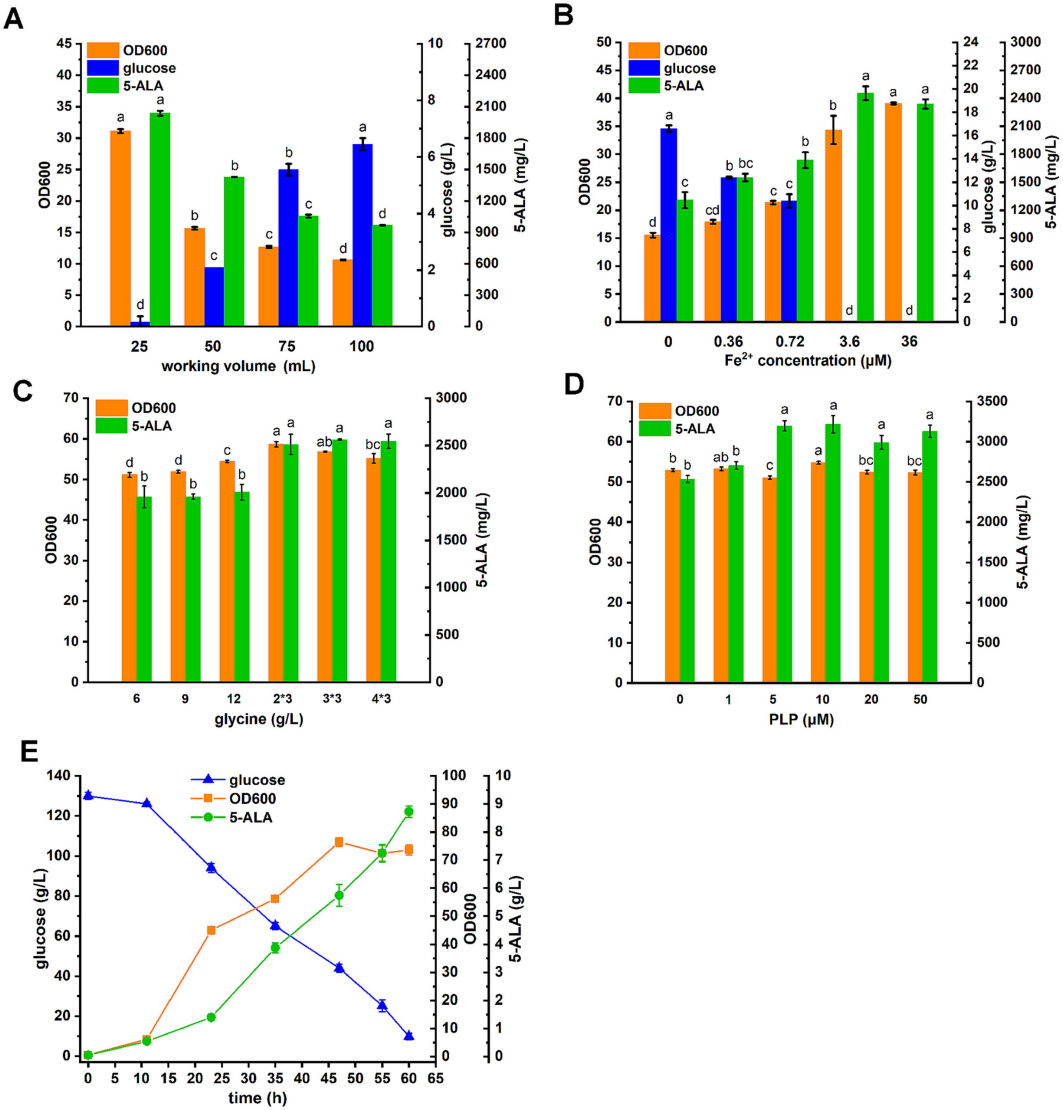
5-ALA fermentation in *C. glutamicum*. **(A)** Effect of working volume on 5-ALA production. Fermentations were performed in 250 mL flasks. **(B)** Effect of Fe^2+^ on 5-ALA production. **(C)** Effect of glycine addition on 5-ALA production. Glycine was added all at once or every 12 h (3 times total for 36 h). **(D)** Effect of PLP on 5-ALA production. **(E)** Prolonged batch fermentation in baffled shake flask. Glucose was added at 130 g/L. Results are averages from three independent experiments, with standard deviations indicated by error bars. Different letters above the bars indicate significant differences (*p* < 0.05).

The capacity of the D4,7,18 mutant for 5-ALA synthesis was tested in the GRAS organism *C. glutamicum* CgS1, which is capable of accumulating the 5-ALA precursor succinyl-CoA (Yang et al., 2016). To balance cell growth and 5-ALA production, the dissolved oxygen (DO) level and Fe^2+^ concentration were optimized. As previous studies have shown, both DO level and Fe^2+^ concentration affect the downstream metabolism of 5-ALA, and thus, influence its accumulation (Yu et al., 2015). The DO level was adjusted by changing the working volume in the 250 mL flask. As a result, both biomass and 5-ALA titer increased as the working volume decreased (Figure 6A). After 36 h, all 30 g/L of glucose had been consumed in the 25 mL set (with a working volume of 25 mL in a 250 mL flask). In addition to the DO level, Fe^2+^ was also essential for cell growth and had a significant effect on both biomass and 5-ALA levels (Figure 6B). As shown, 3.6 μM of Fe^2+^ was sufficient for 5-ALA production in this study.

Next, the amount and mode of precursor glycine addition were investigated. Glycine was added either all at once or every 12 h (three times in total over 36 h) at different amounts (Figure 6C). As a result, when glycine was added at intervals, more 5-ALA accumulated. It appeared that adding 2 g/L of glycine every 12 h was sufficient. Following the optimization of glycine addition, the effect of PLP, the cofactor of ALAS, on 5-ALA production was tested. As a result, the addition of 5 μM PLP increased the 5-ALA titer by 26% compared to the negative control (without PLP addition) and appeared to be sufficient (Figure 6D).

Based on the aforementioned results, prolonged batch fermentation was carried out using baffled flasks to maintain a high DO level by adding 130 g/L of glucose to CGXII medium (Figure 6E). Given the strong aerobic respiration, 0.01% defoaming agent was added. As a result, 8.72 g/L of 5-ALA was produced over 60 h. Organic acid byproducts and glutamate in the fermentation broth were also analyzed. Glutamate was measured at 1.5 g/L, and except for 0.4 g/L of acetate, no other organic acid byproducts were detected, likely due to the complete oxidation of carbon sources resulting from the high DO.

## 4 Discussion

To improve ALAS, the key enzyme for 5-ALA biosynthesis, a growth-coupled selection strategy was developed in this study. *E. coli* DH5α ΔhemA, which is auxotrophic for 5-ALA, was used as the selection strain. When ALAS mutants were heterologously introduced, they rescued the growth deficiency in an enzymatic activity-dependent manner, enabling high-throughput screening of ALAS. At the same time, potential inactive mutants could be excluded.

Plate screening is a low-cost, easy-to-operate method in which potential mutants are selected by simply choosing colonies with larger sizes (Kramer et al., 2020; Calzadiaz-Ramirez et al., 2020). To increase screening efficiency, it is crucial to fine-tune the selection parameters to rule out false positives and ensure a sufficient phenotype (Rix et al., 2020). In this study, the complementation of the selection strain was influenced by both the expression level and enzymatic activity of ALAS mutants. By decreasing the expression level of ALAS (via basal, leaky expression by removing the inducer IPTG), the impact of the enzymatic activity became more pronounced.

The *hemA* gene was mutated either by error-prone PCR or by targeting the active-site loop of ALAS, which regulates the rate-limiting step of product dissociation (Stojanovski et al., 2019). During shake-flask cultivation, biomass accumulation did not always correlate with 5-ALA titers (Figure 2C), likely due to IPTG and glycine supplementation or the higher diffusion coefficient in liquid compared to solid medium.

Color differences among the ALAS variants, primarily arising from downstream porphyrin derivatives, were difficult to discern by the naked eye (Figure 3A). Although porphyrin fluorescence has been used to identify hyperactive ALAS mutants (Lendrihas et al., 2010), our results show that colony color screening can be challenging, whereas growth-coupled plate screening provides a more robust alternative. This underscores the importance of selection parameters in defining the sensitivity and threshold of the method.

Six mutants were screened, with mutant 4 showing the greatest improvement in activity (27.17%). Although mutants 7 and 15 exhibited higher expression levels, they were not the most effective. A previous study reported that the correlation between ALAS activity and 5-ALA production was not strictly positive and suggested that moderate ALAS expression is optimal (Chen et al., 2020). Given the importance of ALAS in multiple physiological processes, the primary selective pressure on this enzyme may favor strict regulation rather than maximal catalytic speed.

To further improve ALAS, combinatorial mutations were constructed. As a result, the triple mutant D4,7,18 produced 1.18-fold higher levels of 5-ALA than the wild type, along with a 67.41% increase in enzymatic activity (Table 3). Enzymatic analysis suggested that the enhanced activity of D4,7,18 was partially due to enhanced PLP binding and a decreased *K*_m_ for glycine. PLP binding is considered the initial step in ALAS catalysis; thus, the D4,7,18 mutation may increase enzyme activity by reinforcing this step (Stojanovski et al., 2019). Previously, the thermostability and hemin inhibition of an *R. palustris*-derived ALAS were reported to be influenced by histidine residues (Tan et al., 2019). Two improved mutants, H29R and H15K, were obtained through computer-aided rational design. In another study, Ikushiro et al. engineered an ALAS from *C. crescentus* by substituting the heme-binding site residues H340 or C398 with glycine, which alleviated heme inhibition (Ikushiro et al., 2018). Additionally, cysteine-targeted mutations were employed to overcome heme inhibition (He et al., 2022). It is foreseeable that the combination of random mutagenesis and structure-based rational design will further enhance enzyme activity in the future.

The fermentation optimization results highlighted the importance of dissolved oxygen, with increased dissolved oxygen found to be beneficial for 5-ALA accumulation. As reported previously, one of the most challenging obstacles for high 5-ALA production is the oxidative stress caused by the accumulation of 5-ALA and downstream metabolites (Zhu et al., 2019). In this study, it appeared that the accumulation of 5-ALA appeared to be positively correlated with DO, in contrast to previous reports (Chen et al., 2020; Miscevic et al., 2021). One possible explanation is that the strain used in this study accumulated succinyl-CoA exclusively via the oxidative branch, due to the interruption of the TCA cycle caused by *sucCD* deletion.

The addition of glycine has previously been reported to incur growth inhibition (Feng et al., 2016). However, biomass increased slightly when glycine was added all at once. This suggests that the added glycine may have been more extensively utilized for protein synthesis under these conditions. In the 5-L fermenter, it seemed that more glycine was required, possibly due to the significantly increased cell density (Chen et al., 2020; Wang W. et al., 2024). As an essential cofactor, 5 μM PLP appeared to be sufficient in this study, compared with 30 μM reported in a previous study (Shih et al., 2021).

Since porphyrin compound formation decreases 5-ALA accumulation, it would be beneficial to precisely tune the downstream pathway to balance 5-ALA production and cellular respiration. On the other hand, precise multistage control of DO and pH in a fermenter may also stabilize product formation (Choi et al., 2022). To drive the synthetic reaction forward and reduce metabolic inhibition, various characterized 5-ALA exporters could be introduced in the future (Wang W. et al., 2024). Furthermore, engineering the endogenous glycine biosynthesis pathway may help simplify the production process and reduce costs (Zou et al., 2017).

## 5 Conclusion

In summary, a growth-coupled selection strategy was developed to enhance ALAS activity in this study. The best mutant showed a 67.41% improvement in enzymatic activity. When expressed in *C. glutamicum*, the optimized strain produced 8.72 g/L of 5-ALA, with minimal accumulation of organic acid byproducts. The selection strategy presented here could serve as a useful approach for optimizing other essential metabolic pathways.

## Data Availability

The original contributions presented in the study are included in the article/Supplementary material, further inquiries can be directed to the corresponding author.

## References

[ref1] Aiguo Z. Meizhi Z. (2019). Production of 5-aminolevulinic acid from glutamate by overexpressing *HemA1* and *pgr7* from *Arabidopsis thaliana* in *Escherichia coli*. World J. Microbiol. Biotechnol. 35:175. doi: 10.1007/s11274-019-2750-6, PMID: 31673852

[ref2] Bradford M. M. (1976). A rapid and sensitive method for the quantitation of microgram quantities of protein utilizing the principle of protein-dye binding. Anal. Biochem. 72, 248–254. doi: 10.1016/0003-2697(76)90527-3, PMID: 942051

[ref3] Calzadiaz-Ramirez L. Calvó-Tusell C. Stoffel G. M. Lindner S. N. Osuna S. Erb T. J. . (2020). *In vivo* selection for formate dehydrogenases with high efficiency and specificity toward NADP+. ACS Catal. 10, 7512–7525. doi: 10.1021/acscatal.0c01487, PMID: 32733773 PMC7384739

[ref4] Chen J. Wang Y. Guo X. Rao D. Zhou W. Zheng P. . (2020). Efficient bioproduction of 5-aminolevulinic acid, a promising biostimulant and nutrient, from renewable bioresources by engineered *Corynebacterium glutamicum*. Biotechnol. Biofuels 13:41. doi: 10.1186/s13068-020-01685-0, PMID: 32175008 PMC7063817

[ref5] Choi K. R. Yu H. E. Lee H. Lee S. Y. (2022). Improved production of heme using metabolically engineered *Escherichia coli*. Biotechnol. Bioeng. 119, 3178–3193. doi: 10.1002/bit.28194, PMID: 35892195

[ref6] Cui Z. Jiang Z. Zhang J. Zheng H. Jiang X. Gong K. . (2019). Stable and efficient biosynthesis of 5-aminolevulinic acid using plasmid-free *Escherichia coli*. J. Agric. Food Chem. 67, 1478–1483. doi: 10.1021/acs.jafc.8b06496, PMID: 30644739

[ref7] Dailey H. A. Dailey T. A. Gerdes S. Jahn D. Jahn M. O'Brian M. R. . (2017). Prokaryotic heme biosynthesis: multiple pathways to a common essential product. Microbiol. Mol. Biol. Rev. 81:e00048-16. doi: 10.1128/mmbr.00048-16, PMID: 28123057 PMC5312243

[ref8] Datsenko K. A. Wanner B. L. (2000). One-step inactivation of chromosomal genes in *Escherichia coli* K-12 using PCR products. Proc. Natl. Acad. Sci. USA 97, 6640–6645. doi: 10.1073/pnas.120163297, PMID: 10829079 PMC18686

[ref9] Delgado J. Radusky L. G. Cianferoni D. Serrano L. (2019). FoldX 5.0: working with RNA, small molecules and a new graphical interface. Bioinformatics 35, 4168–4169. doi: 10.1093/bioinformatics/btz184, PMID: 30874800 PMC6792092

[ref10] Feng L. Zhang Y. Fu J. Mao Y. Chen T. Zhao X. . (2016). Metabolic engineering of *Corynebacterium glutamicum* for efficient production of 5-aminolevulinic acid. Biotechnol. Bioeng. 113, 1284–1293. doi: 10.1002/bit.25886, PMID: 26616115

[ref11] Ge F. Li X. Ge Q. Zhu D. Li W. Shi F. . (2021). Modular control of multiple pathways of *Corynebacterium glutamicum* for 5-aminolevulinic acid production. AMB Express 11:179. doi: 10.1186/s13568-021-01335-0, PMID: 34958433 PMC8712284

[ref12] He G. Jiang M. Cui Z. Sun X. Chen T. Wang Z. (2022). Construction of 5-aminolevulinic acid synthase variants by cysteine-targeted mutation to release heme inhibition. J. Biosci. Bioeng. 134, 416–423. doi: 10.1016/j.jbiosc.2022.07.019, PMID: 36089467

[ref13] Ikushiro H. Nagami A. Takai T. Sawai T. Shimeno Y. Hori H. . (2018). Heme-dependent inactivation of 5-aminolevulinate synthase from *Caulobacter crescentus*. Sci. Rep. 8:14228. doi: 10.1038/s41598-018-32591-z, PMID: 30242198 PMC6154995

[ref14] Jeong J. Y. Yim H. S. Ryu J. Y. Lee H. S. Lee J. H. Seen D. S. . (2012). One-step sequence- and ligation-independent cloning as a rapid and versatile cloning method for functional genomics studies. Appl. Environ. Microbiol. 78, 5440–5443. doi: 10.1128/AEM.00844-12, PMID: 22610439 PMC3416421

[ref15] Jiang M. Hong K. Mao Y. Ma H. Chen T. Wang Z. (2022). Natural 5-aminolevulinic acid: sources, biosynthesis, detection and applications. Front. Bioeng. Biotechnol. 10:841443. doi: 10.3389/fbioe.2022.841443, PMID: 35284403 PMC8913508

[ref16] Jun Y. Li Z. Weiqi F. Yijun L. Jianping L. Peilin C. (2013). Improved 5-aminolevulinic acid production with recombinant *Escherichia coli* by a short-term dissolved oxygen shock in fed-batch fermentation. Chin. J. Chem. Eng. 21, 1291–1295. doi: 10.1016/S1004-9541(13)60627-8

[ref17] Kang Z. Wang Y. Gu P. Wang Q. Qi Q. (2011). Engineering *Escherichia coli* for efficient production of 5-aminolevulinic acid from glucose. Metab. Eng. 13, 492–498. doi: 10.1016/j.ymben.2011.05.003, PMID: 21620993

[ref18] Kramer L. Le X. Rodriguez M. Wilson M. A. Guo J. Niu W. (2020). Engineering carboxylic acid reductase (CAR) through a whole-cell growth-coupled NADPH recycling strategy. ACS Synth. Biol. 9, 1632–1637. doi: 10.1021/acssynbio.0c00290, PMID: 32589835

[ref19] Lendrihas T. Hunter G. A. Ferreira G. C. (2010). Targeting the active site gate to yield hyperactive variants of 5-aminolevulinate synthase. J. Biol. Chem. 285, 13704–13711. doi: 10.1074/jbc.M109.074237, PMID: 20194506 PMC2859533

[ref20] Li Z. Deng Y. Yang G. Y. (2023). Growth-coupled high throughput selection for directed enzyme evolution. Biotechnol. Adv. 68:108238. doi: 10.1016/j.biotechadv.2023.108238, PMID: 37619825

[ref21] Lin J. Fu W. Cen P. (2009). Characterization of 5-aminolevulinate synthase from *Agrobacterium radiobacter*, screening new inhibitors for 5-aminolevulinate dehydratase from *Escherichia coli* and their potential use for high 5-aminolevulinate production. Bioresour. Technol. 100, 2293–2297. doi: 10.1016/j.biortech.2008.11.008, PMID: 19095441

[ref22] Liu X. X. Wang L. Wang Y. J. Cai L. L. (2010). D-glucose enhanced 5-aminolevulinic acid production in recombinant *Escherichia coli* culture. Appl. Biochem. Biotechnol. 160, 822–830. doi: 10.1007/s12010-009-8608-x, PMID: 19381488

[ref23] Luo Z. Pan F. Zhu Y. Du S. Yan Y. Wang R. . (2022). Synergistic improvement of 5-aminolevulinic acid production with synthetic scaffolds and system pathway engineering. ACS Synth. Biol. 11, 2766–2778. doi: 10.1021/acssynbio.2c00157, PMID: 35939037

[ref24] Miscevic D. Mao J. Y. Kefale T. Abedi D. Moo-Young M. Perry Chou C. (2021). Strain engineering for high-level 5-aminolevulinic acid production in *Escherichia coli*. Biotechnol. Bioeng. 118, 30–42. doi: 10.1002/bit.27547, PMID: 32860420

[ref25] Morris G. M. Huey R. Lindstrom W. Sanner M. F. Belew R. K. Goodsell D. S. . (2009). AutoDock4 and AutoDockTools4: automated docking with selective receptor flexibility. J. Comput. Chem. 30, 2785–2791. doi: 10.1002/jcc.21256, PMID: 19399780 PMC2760638

[ref26] Rix G. Watkins-Dulaney E. J. Almhjell P. J. Boville C. E. Arnold F. H. Liu C. C. (2020). Scalable continuous evolution for the generation of diverse enzyme variants encompassing promiscuous activities. Nat. Commun. 11:5644. doi: 10.1038/s41467-020-19539-6, PMID: 33159067 PMC7648111

[ref27] Schake P. Bolz S. N. Linnemann K. Schroeder M. (2025). PLIP 2025: introducing protein–protein interactions to the protein-ligand interaction profiler. Nucleic Acids Res. 53, W463–W465. doi: 10.1093/nar/gkaf361, PMID: 40347107 PMC12230730

[ref28] Shih I.-T. Yi Y.-C. Ng I.-S. (2021). Plasmid-free system and modular design for efficient 5-aminolevulinic acid production by engineered *Escherichia coli*. Appl. Biochem. Biotechnol. 193, 2858–2871. doi: 10.1007/s12010-021-03571-3, PMID: 33860878

[ref29] Stojanovski B. M. Hunter G. A. Na I. Uversky V. N. Jiang R. H. Y. Ferreira G. C. (2019). 5-Aminolevulinate synthase catalysis: the catcher in heme biosynthesis. Mol. Genet. Metab. 128, 178–189. doi: 10.1016/j.ymgme.2019.06.003, PMID: 31345668 PMC6908770

[ref30] Tan Z. Zhao J. Chen J. Rao D. Zhou W. Chen N. . (2019). Enhancing thermostability and removing hemin inhibition of *Rhodopseudomonas palustris* 5-aminolevulinic acid synthase by computer-aided rational design. Biotechnol. Lett. 41, 181–191. doi: 10.1007/s10529-018-2627-z, PMID: 30498972

[ref31] Wang Q. Jia M. Li H. Li Q. Zhang J. Su T. . (2024). Design of a genetically encoded biosensor for high-throughput screening and engineering 5-aminolevulinic acid hyper-producing *Escherichia coli*. ACS Sustain. Chem. Eng. 12, 4846–4857. doi: 10.1021/acssuschemeng.3c06991

[ref32] Wang W. Xiang Y. Yin G. Hu S. Cheng J. Chen J. . (2024). Construction of 5-aminolevulinic acid microbial cell factories through identification of novel synthases and metabolic pathway screens and transporters. J. Agric. Food Chem. 72, 8006–8017. doi: 10.1021/acs.jafc.4c00903, PMID: 38554273

[ref33] Xie L. Hall D. Eiteman M. Altman E. (2003). Optimization of recombinant aminolevulinate synthase production in *Escherichia coli* using factorial design. Appl. Microbiol. Biotechnol. 63, 267–273. doi: 10.1007/s00253-003-1388-2, PMID: 14661117

[ref34] Yang P. Liu W. Cheng X. Wang J. Wang Q. Qi Q. (2016). A new strategy for production of 5-aminolevulinic acid in recombinant *Corynebacterium glutamicum* with high yield. Appl. Environ. Microbiol. 82, 2709–2717. doi: 10.1128/AEM.00224-16, PMID: 26921424 PMC4836415

[ref35] Yu X. Jin H. Liu W. Wang Q. Qi Q. (2015). Engineering *Corynebacterium glutamicum* to produce 5-aminolevulinic acid from glucose. Microb. Cell Factories 14:183. doi: 10.1186/s12934-015-0364-8, PMID: 26577071 PMC4650169

[ref36] Zhang L. Chen J. Chen N. Sun J. Zheng P. Ma Y. (2013). Cloning of two 5-aminolevulinic acid synthase isozymes HemA and HemO from *Rhodopseudomonas palustris* with favorable characteristics for 5-aminolevulinic acid production. Biotechnol. Lett. 35, 763–768. doi: 10.1007/s10529-013-1143-4, PMID: 23338702

[ref37] Zhang C. Li Y. Zhu F. Li Z. Lu N. Li Y. . (2020). Metabolic engineering of an auto-regulated *Corynebacterium glutamicum* chassis for biosynthesis of 5-aminolevulinic acid. Bioresour. Technol. 318:124064. doi: 10.1016/j.biortech.2020.124064, PMID: 32905949

[ref38] Zhang J. Weng H. Zhou Z. Du G. Kang Z. (2019). Engineering of multiple modular pathways for high-yield production of 5-aminolevulinic acid in *Escherichia coli*. Bioresour. Technol. 274, 353–360. doi: 10.1016/j.biortech.2018.12.004, PMID: 30537593

[ref39] Zhao J. Bo T. Wu Y. Geng Z. Zhao J. Wu K. . (2025). Engineering *Corynebacterium glutamicum* for the production of 5-aminolevulinic acid under microaerobic conditions guided by a genome-scale metabolic network. J. Agric. Food Chem. 73, 12809–12820. doi: 10.1021/acs.jafc.4c10853, PMID: 40365842

[ref40] Zhou H. Zhang C. Li Z. Xia M. Li Z. Wang Z. . (2024). Systematic development of a highly efficient cell factory for 5-aminolevulinic acid production. Trends Biotechnol. 42, 1479–1502. doi: 10.1016/j.tibtech.2024.06.004, PMID: 39112275

[ref41] Zhu C. Chen J. Wang Y. Wang L. Guo X. Chen N. . (2019). Enhancing 5-aminolevulinic acid tolerance and production by engineering the antioxidant defense system of *Escherichia coli*. Biotechnol. Bioeng. 116, 2018–2028. doi: 10.1002/bit.26981, PMID: 30934113

[ref42] Zou Y. Chen T. Feng L. Zhang S. Xing D. Wang Z. (2017). Enhancement of 5-aminolevulinic acid production by metabolic engineering of the glycine biosynthesis pathway in *Corynebacterium glutamicum*. Biotechnol. Lett. 39, 1369–1374. doi: 10.1007/s10529-017-2362-x, PMID: 28536938

