# Supplementary material for: Growth-coupled selection of 5-aminolevulinic acid synthetase mutants for 5-aminolevulinic acid biosynthesis in Corynebacterium glutamicum
Source: Front Microbiol. 2025 Nov 18;16:1717625. doi: 10.3389/fmicb.2025.1717625 (PMC12746484; doi:10.3389/fmicb.2025.1717625)
Supplement: Supplementary file 1 [file Table_1.DOCX]

Supplementary Material

## 1 Supplementary Figures


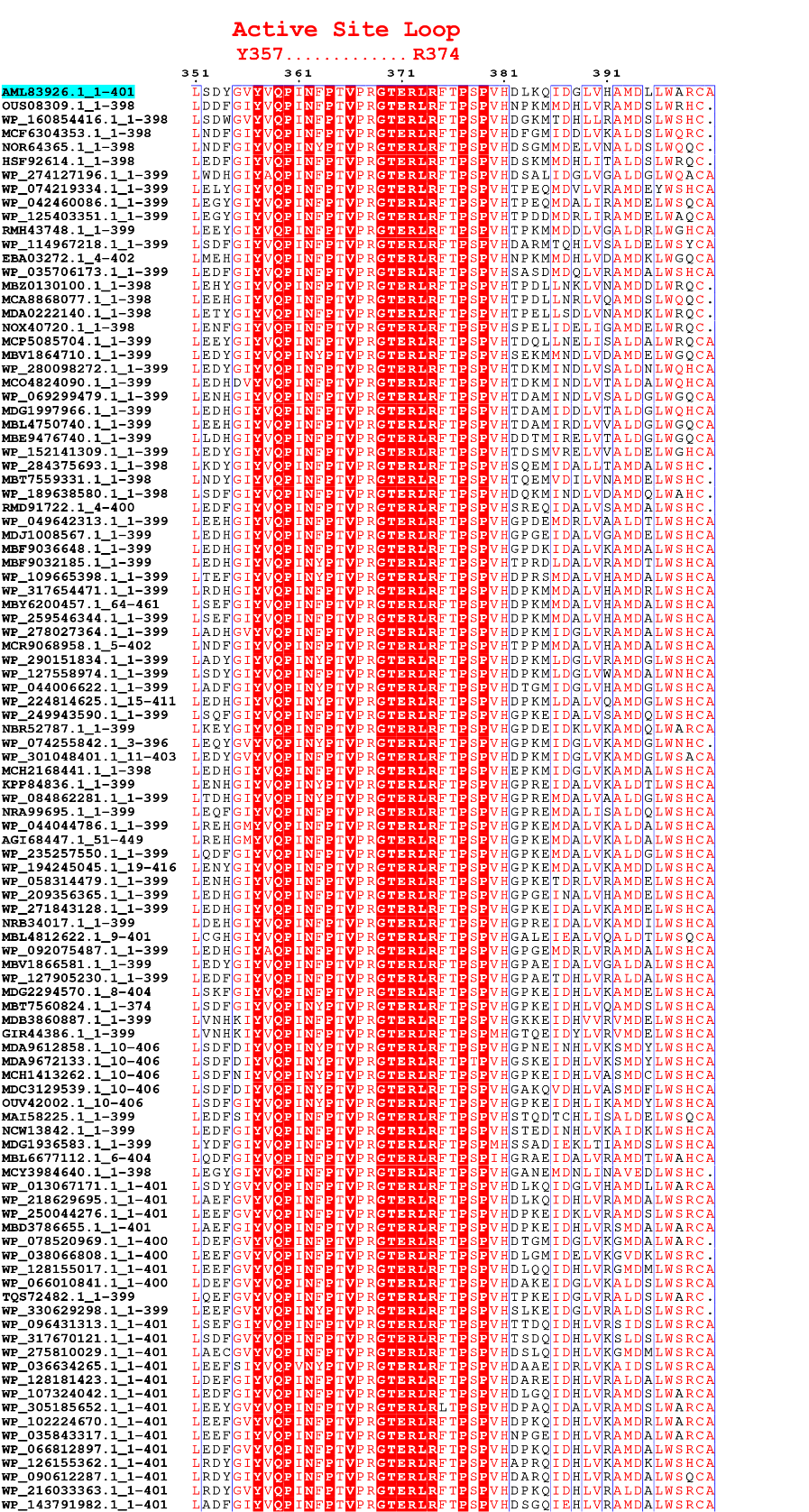


(Supplementary Figure 1 continued)


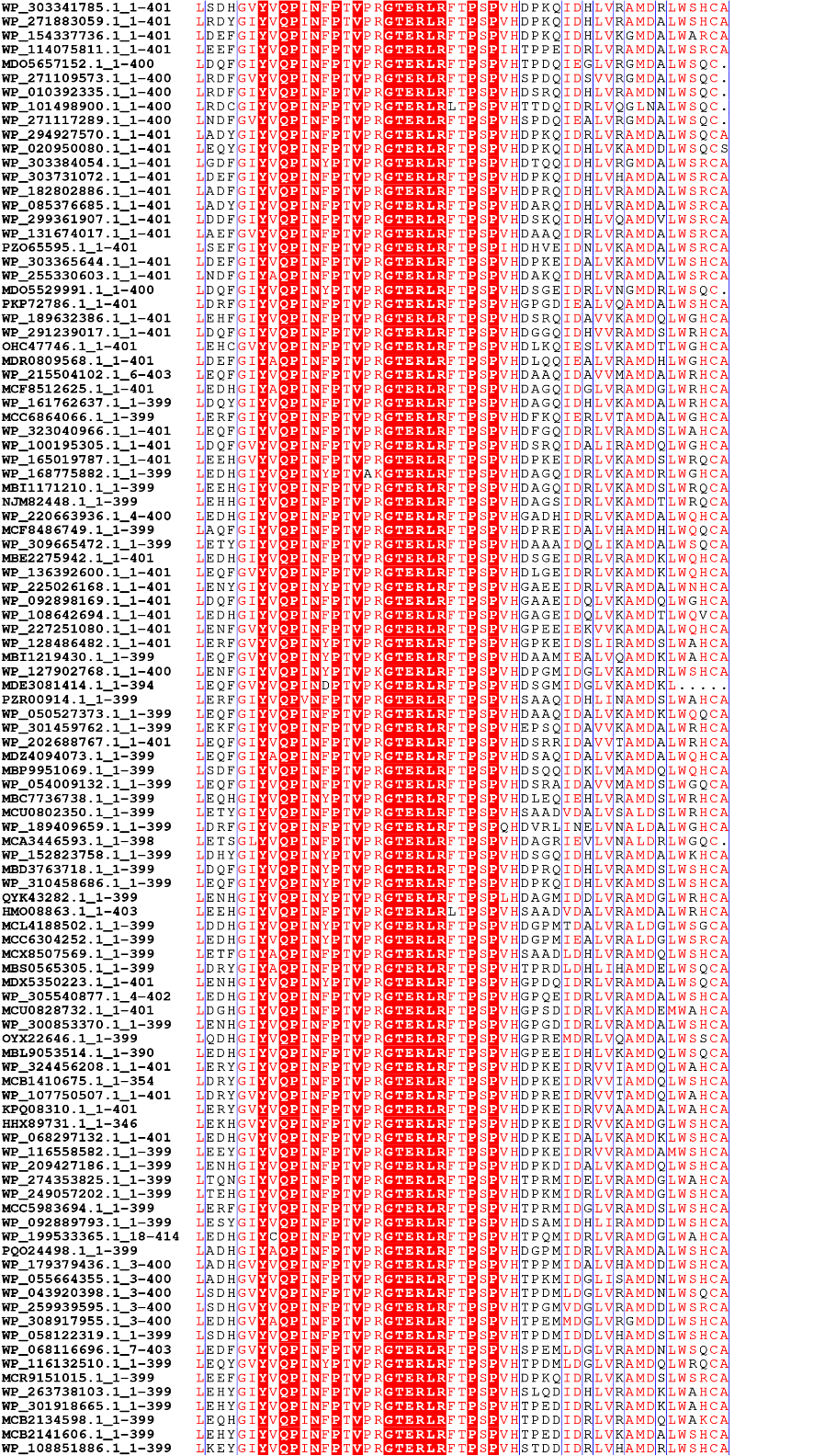


(Supplementary Figure 1 continued)


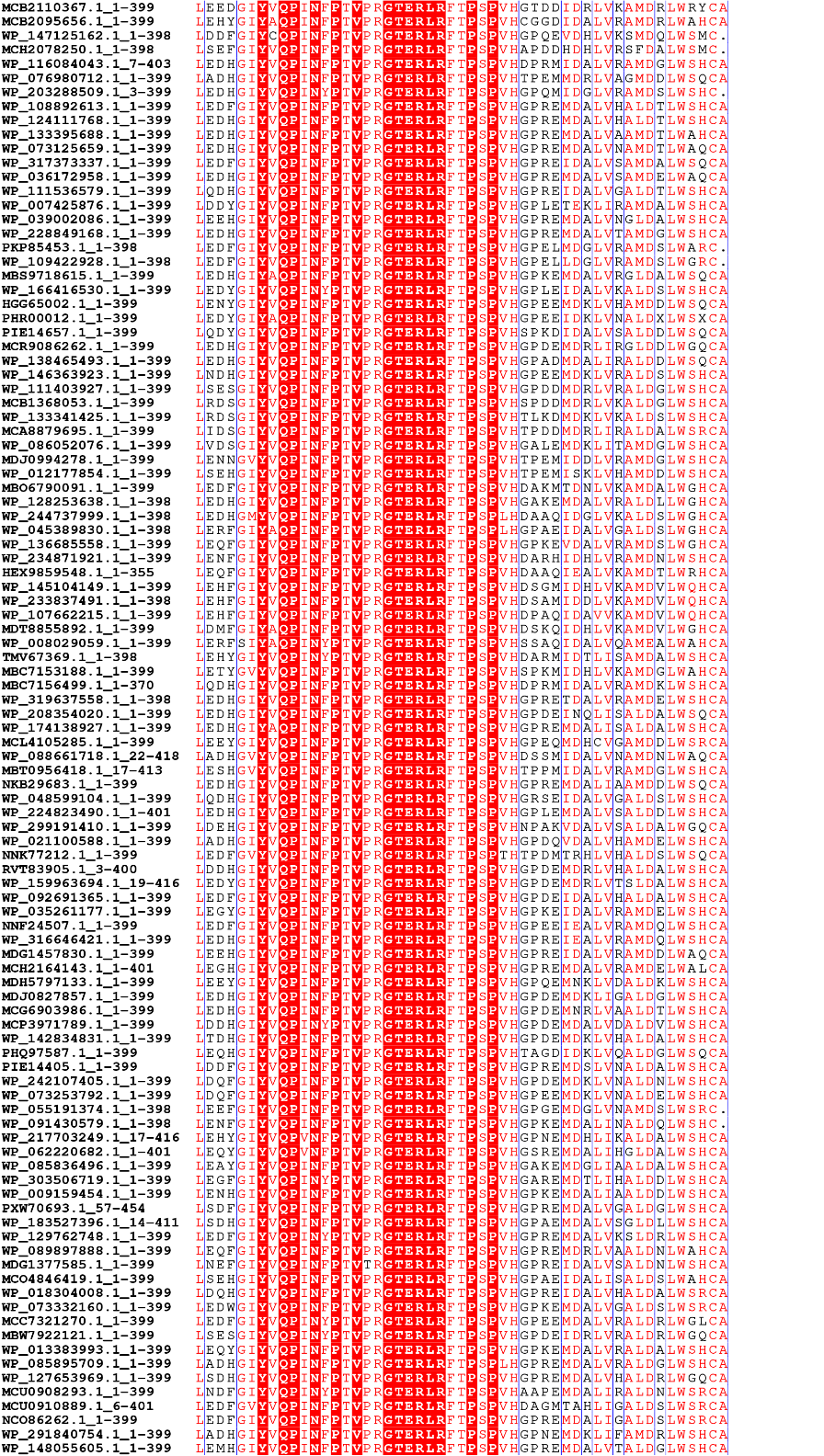


(Supplementary Figure 1 continued)


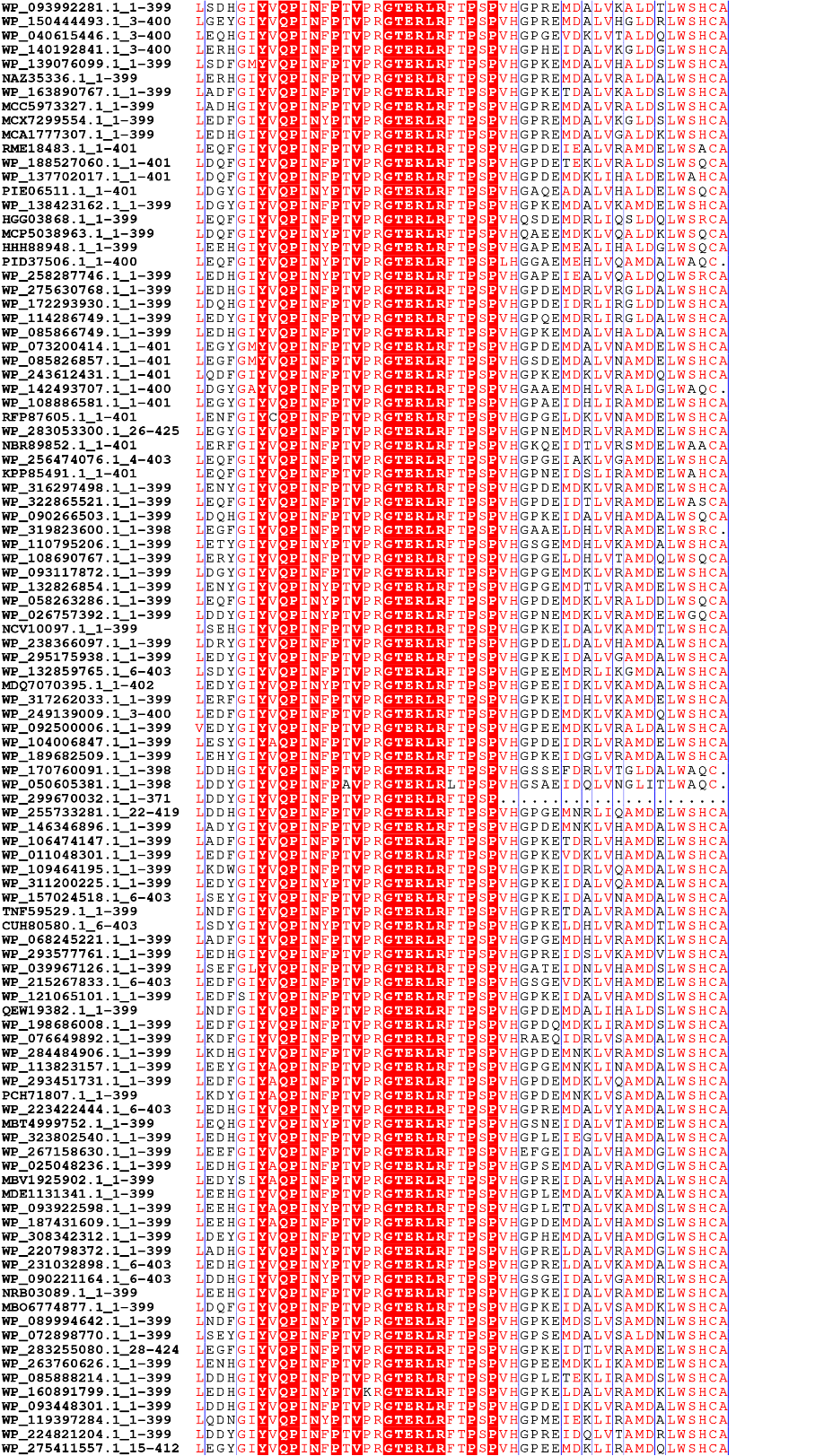


(Supplementary Figure 1 continued)


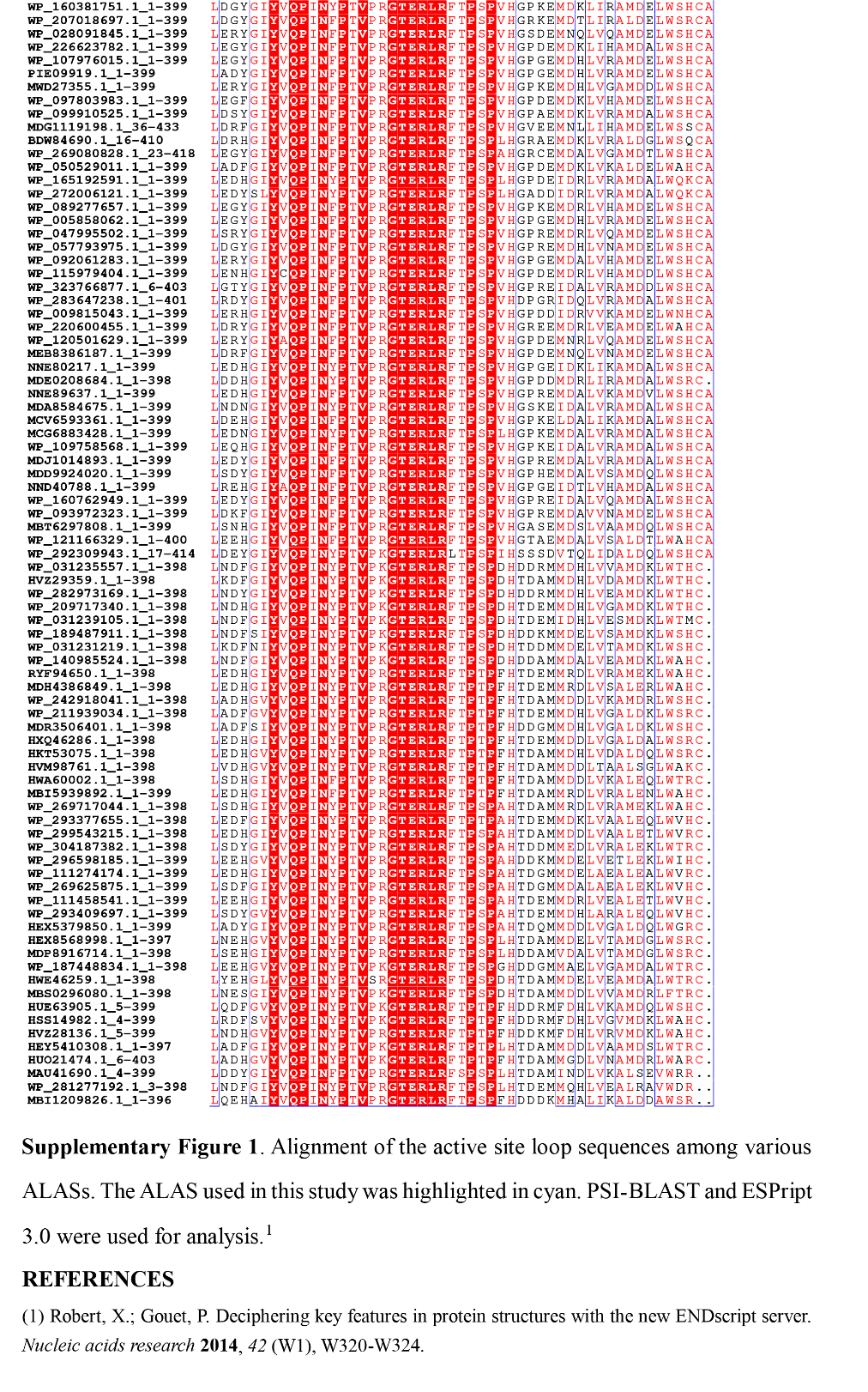


**Supplementary Figure 1.** Alignment of the active site loop sequences among various ALASs. The ALAS used in this study was highlighted in cyan. PSI-BLAST and ESPript 3.0 were used for analysis (Robert and Gouet, 2014).

## 2 Supplementary Tables

**Supplementary Table 1. Oligonucleotides used in this study.**

| Oligonucleotides | Sequence (5' to 3') |
| --- | --- |
| hemA-F | AACGTGTATCGTTTTCGCCGGATAAGCTCGATCAGGCGCTTGACAGCCTGTGTAGGCTGGAGCTGCTTC |
| hemA-R | ACGGGCGGCCTGTTGAAGTGATTTCGTTGGCGCATGGATCAAGCGGTTAATGGGAATTAGCCATGGTCC |
| sbmF | CACACAGGAAACAGACCATGGAATTCGAATTAAGGAGATATACATATG |
| sbmR | GCCTGCAGGTCGACTCTAGAGGATCCTTAGGCACAGCGAGCCCAGAG |
| mSB-F1 | CTTTCTGACTACGGCGTCTAT**GYT**CAACCTATCAAT**TWC**CCAACCGTG**NNKMRM**GGTACCGAGCGTCTGCGTTTTACC* |
| mSB-R1 | TTGTCCAGATAGCCCAGTA |
| mSB-F2 | TACTGGGCTATCTGGACAAGGGAAAACGCAAGCGCAAAGAG |
| mSB-R2 | ATAGACGCCGTAGTCAGAAAGCAACATATCGGAAACTGCTTTG |
| test-F | GCTCGTATAATGTGTGGAAT |
| test-R | GCGTTCTGATTTAATCTGTATC |
| c-f | CTACATCGATGAAGTGCACGCTGTGGGCATGTATGGTCC |
| c-r | GCGTGCACTTCATCGATGTAG |
| c-f2 | GAAGCTCTTGAGGCTGTGGGTGCTGGTTCCGGCGGTACC |
| c-r2 | CCACAGCCTCAAGAGCTTC |
| mSB-F3 | GCTTTCTGACTA CGGCGTCTAT |

*Y=C/T, N=A/T/C/G, K=G/T, M=A/C, R=A/G.

**Supplementary** **Table 2. Primary screen of ALAS mutants.**

| Strain^a^ | Redesignated name^b^ | OD600 | 5-ALA (mg/L) | 5-ALA/OD |
| --- | --- | --- | --- | --- |
| R1 | - | 3.08 | 32.23 | 10.46 |
| R2 | - | 3.27 | 18.80 | 5.75 |
| R3 | - | 3.16 | 22.62 | 7.16 |
| R4 | - | 3.41 | 24.17 | 7.09 |
| R5 | - | 3.74 | 29.44 | 7.87 |
| R6 | - | 3.08 | 28.10 | 9.12 |
| R7 | - | 2.9 | 24.90 | 8.59 |
| R8 | - | 3.42 | 30.79 | 9.00 |
| R9 | - | 3.61 | 24.28 | 6.72 |
| R10 | - | 3.61 | 28.00 | 7.76 |
| R11 | - | 3.68 | 26.65 | 7.24 |
| R12 | - | 3.7 | 25.31 | 6.84 |
| R13 | 1 | 3.71 | 56.41 | 15.20 |
| R14 | - | 3.37 | 17.98 | 5.33 |
| R15 | - | 3.43 | 32.75 | 9.55 |
| R16 | - | 2.96 | 27.69 | 9.35 |
| R17 | - | 3.49 | 28.51 | 8.17 |
| R18 | - | 3.61 | 31.20 | 8.64 |
| R19 | - | 3.58 | 38.95 | 10.88 |
| R20 | - | 3.2 | 26.76 | 8.36 |
| R21 | - | 3.72 | 36.67 | 9.86 |
| R22 | - | 3.56 | 24.79 | 6.96 |
| R23 | 2 | 3.07 | 38.93 | 12.68 |
| R24 | - | 3.01 | 29.03 | 9.64 |
| R25 | - | 3.37 | 23.24 | 6.90 |
| R26 | - | 3.64 | 34.92 | 9.59 |
| R27 | - | 3.53 | 16.22 | 4.59 |
| R28 | - | 3.51 | 34.40 | 9.80 |
| R29 | - | 3.42 | 32.44 | 9.48 |
| R30 | - | 3.32 | 26.14 | 7.87 |
| R31 | - | 3.26 | 15.08 | 4.63 |
| R32 | 3 | 3.48 | 43.80 | 12.59 |
| R33 | - | 3.53 | 16.63 | 4.71 |
| R34 | - | 3.57 | 17.77 | 4.98 |
| R35 | - | 3.03 | 17.15 | 5.66 |
| R36 | - | 3.56 | 15.91 | 4.47 |
| R37 | - | 3.04 | 16.94 | 5.57 |
| R38 | 4 | 3.16 | 61.57 | 19.48 |
| R39 | - | 3.36 | 30.37 | 9.04 |
| R40 | 5 | 3.06 | 43.80 | 14.31 |
| R41 | - | 3.61 | 22.73 | 6.30 |
| R42 | - | 3.56 | 18.50 | 5.20 |
| R43 | - | 2.77 | 12.71 | 4.59 |
| R44 | - | 3.55 | 21.38 | 6.02 |
| R45 | - | 2.94 | 13.64 | 4.64 |
| R46 | - | 3.72 | 19.94 | 5.36 |
| R47 | - | 2.72 | 14.05 | 5.17 |
| R48 | - | 2.45 | 12.81 | 5.23 |
| R49 | - | 3.59 | 14.98 | 4.17 |
| R50 | - | 3.34 | 27.18 | 8.14 |
| R51 | - | 3.6 | 38.41 | 10.67 |
| R52 | - | 3.44 | 16.07 | 4.67 |
| R53 | - | 3.54 | 37.4 | 10.56 |
| R54 | - | 3.36 | 39.16 | 11.65 |
| R55 | - | 3.78 | 17.94 | 4.75 |
| R56 | - | 3.65 | 18.78 | 5.15 |
| R57 | - | 3.32 | 37.74 | 11.37 |
| R58 | - | 3.67 | 16.91 | 4.61 |
| R59 | - | 3.76 | 35.27 | 9.38 |
| R60 | - | 3.08 | 22.93 | 7.44 |
| R61 | - | 3.24 | 16.24 | 5.01 |
| R62 | 6 | 3.56 | 48.62 | 13.66 |
| R63 | - | 2.94 | 21.03 | 7.15 |
| R64 | - | 2.88 | 20.75 | 7.20 |
| R65 | 7 | 2.9 | 49.53 | 17.08 |
| R66 | - | 3.37 | 28.89 | 8.57 |
| R67 | - | 3.1 | 29.49 | 9.51 |
| R68 | - | 3.5 | 22.78 | 6.51 |
| R69 | 8 | 2.3 | 36.91 | 16.05 |
| R70 | - | 3.2 | 36.46 | 11.39 |
| R71 | - | 2.51 | 26.32 | 10.49 |
| R72 | - | 2.34 | 24.03 | 10.27 |
| R73 | - | 3.28 | 32.35 | 9.86 |
| R74 | - | 3 | 35.54 | 11.85 |
| R75 | - | 2.99 | 32.17 | 10.76 |
| R76 | - | 3.01 | 34.33 | 11.41 |
| R77 | - | 3.24 | 35.43 | 10.94 |
| R78 | - | 2.81 | 17.45 | 6.21 |
| R79 | - | 2.71 | 31.44 | 11.60 |
| R80 | - | 3.02 | 30.13 | 9.98 |
| R81 | - | 3.39 | 18.3 | 5.40 |
| R82 | - | 3.99 | 30.04 | 7.53 |
| R83 | 9 | 3.71 | 47.67 | 12.85 |
| R84 | - | 3.3 | 17.08 | 5.18 |
| R85 | - | 3.91 | 18.01 | 4.61 |
| R86 | - | 3.2 | 36.1 | 11.28 |
| R87 | - | 3.4 | 27.84 | 8.19 |
| R88 | - | 3.63 | 34.99 | 9.64 |
| R89 | - | 3.34 | 22.54 | 6.75 |
| R90 | - | 2.76 | 23.75 | 8.61 |
| R91 | - | 3.21 | 19.31 | 6.02 |
| R92 | - | 3.35 | 27.01 | 8.06 |
| R93 | 10 | 3.69 | 50.05 | 13.56 |
| R94 | - | 3.54 | 16.84 | 4.76 |
| R95 | - | 3.76 | 30.79 | 8.19 |
| R96 | - | 3.55 | 37.52 | 10.57 |
| R97 | - | 3.84 | 35.97 | 9.37 |
| R98 | - | 4.16 | 30.52 | 7.34 |
| R99 | - | 3.62 | 36.98 | 10.22 |
| R100 | - | 3.58 | 37.47 | 10.47 |
| T1 | - | 2.95 | 22.11 | 7.49 |
| T2 | - | 3.12 | 15.32 | 4.91 |
| T3 | - | 3.71 | 23.45 | 6.32 |
| T4 | - | 3.27 | 31.20 | 9.54 |
| T5 | - | 2.75 | 27.58 | 10.03 |
| T6 | - | 3.66 | 29.86 | 8.16 |
| T7 | 11 | 3.16 | 41.12 | 13.01 |
| T8 | - | 2.68 | 24.08 | 8.98 |
| T9 | - | 3.73 | 16.94 | 4.54 |
| T10 | - | 3.74 | 14.15 | 3.78 |
| T11 | - | 3.2 | 20.25 | 6.33 |
| T12 | - | 3.51 | 17.98 | 5.12 |
| T13 | - | 2.98 | 13.33 | 4.47 |
| T14 | - | 2.76 | 16.94 | 6.14 |
| T15 | - | 3.59 | 18.80 | 5.24 |
| T16 | - | 2.62 | 20.35 | 7.77 |
| T17 | - | 2.87 | 23.07 | 8.04 |
| T18 | - | 3.13 | 13.53 | 4.32 |
| T19 | - | 2.76 | 31.20 | 11.30 |
| T20 | - | 3.55 | 19.90 | 5.61 |
| T21 | - | 3.22 | 24.90 | 7.73 |
| T22 | 12 | 2.74 | 43.80 | 15.99 |
| T23 | 13 | 3.28 | 45.66 | 13.92 |
| T24 | - | 3 | 22.73 | 7.58 |
| T25 | - | 2.62 | 22.93 | 8.75 |
| T26 | - | 3.65 | 12.09 | 3.31 |
| T27 | - | 3.67 | 34.19 | 9.32 |
| T28 | - | 2.77 | 30.10 | 10.87 |
| T29 | - | 2.63 | 21.69 | 8.25 |
| T30 | - | 3.34 | 12.19 | 3.65 |
| T31 | - | 2.91 | 14.15 | 4.86 |
| T32 | - | 3.27 | 22.94 | 7.01 |
| T33 | - | 3.26 | 11.05 | 3.39 |
| T34 | 14 | 2.96 | 48.76 | 16.47 |
| T35 | - | 2.68 | 29.75 | 11.10 |
| T36 | - | 2.94 | 13.53 | 4.60 |
| T37 | - | 2.73 | 15.39 | 5.64 |
| T38 | - | 3.2 | 23.66 | 7.39 |
| T39 | - | 2.87 | 16.12 | 5.62 |
| T40 | - | 3.19 | 11.36 | 3.56 |
| T41 | - | 3.22 | 30.79 | 9.56 |
| T42 | - | 3.64 | 41.43 | 11.38 |
| T43 | - | 3.53 | 13.33 | 3.78 |
| T44 | 15 | 2.67 | 41.43 | 15.52 |
| T45 | - | 3.44 | 15.35 | 4.46 |
| T46 | - | 3.44 | 20.56 | 5.98 |
| T47 | - | 2.84 | 29.89 | 10.53 |
| T48 | - | 3.13 | 23.90 | 7.64 |
| T49 | - | 3.8 | 20.87 | 5.49 |
| T50 | - | 2.99 | 20.56 | 6.88 |
| T51 | - | 3.12 | 23.86 | 7.65 |
| T52 | 16 | 3.14 | 40.19 | 12.80 |
| T53 | - | 2.85 | 15.78 | 5.54 |
| T54 | - | 3.28 | 16.90 | 5.15 |
| T55 | - | 3.55 | 27.89 | 7.86 |
| T56 | - | 3.35 | 23.90 | 7.14 |
| T57 | - | 2.99 | 19.25 | 6.44 |
| T58 | 17 | 3.52 | 45.46 | 12.91 |
| T59 | - | 3.35 | 34.09 | 10.18 |
| T60 | - | 2.71 | 29.46 | 10.87 |
| T61 | - | 2.83 | 22.73 | 8.03 |
| T62 | - | 3.17 | 21.49 | 6.78 |
| T63 | - | 3.12 | 19.84 | 6.36 |
| T64 | - | 3.49 | 40.81 | 11.69 |
| T65 | - | 2.86 | 26.03 | 9.10 |
| T66 | - | 3.79 | 23.35 | 6.16 |
| T67 | - | 3.1 | 34.22 | 11.04 |
| T68 | - | 3.25 | 32.12 | 9.88 |
| T69 | - | 3.54 | 22.73 | 6.42 |
| T70 | - | 3.61 | 22.31 | 6.18 |
| T71 | - | 3.22 | 24.28 | 7.54 |
| T72 | - | 2.92 | 20.25 | 6.93 |
| T73 | - | 3.04 | 21.28 | 7.00 |
| T74 | - | 2.74 | 30.79 | 11.24 |
| T75 | - | 3.21 | 34.71 | 10.81 |
| T76 | - | 2.99 | 27.89 | 9.33 |
| T77 | - | 3.79 | 40.70 | 10.74 |
| T78 | - | 3.17 | 32.13 | 10.14 |
| T79 | - | 3.2 | 21.49 | 6.72 |
| T80 | - | 3.35 | 32.75 | 9.78 |
| T81 | - | 3.21 | 30.89 | 9.62 |
| T82 | - | 3.01 | 18.39 | 6.11 |
| T83 | - | 2.8 | 20.15 | 7.19 |
| T84 | - | 2.62 | 17.98 | 6.86 |
| T85 | - | 3.58 | 18.18 | 5.08 |
| T86 | - | 3.34 | 37.09 | 11.10 |
| T87 | - | 2.65 | 31.30 | 11.81 |
| T88 | - | 3.51 | 36.88 | 10.51 |
| T89 | - | 3.69 | 24.48 | 6.63 |
| T90 | 18 | 3.47 | 54.13 | 15.60 |
| T91 | - | 3.4 | 19.02 | 5.60 |
| T92 | - | 2.79 | 20.11 | 7.21 |
| T93 | - | 3.39 | 24.28 | 7.16 |
| T94 | 19 | 3.17 | 42.25 | 13.33 |
| T95 | - | 2.88 | 25.41 | 8.82 |
| T96 | - | 3.75 | 30.87 | 8.23 |
| T97 | - | 3.13 | 19.00 | 6.07 |
| T98 | - | 3.27 | 20.25 | 6.19 |
| T99 | 20 | 3.52 | 51.65 | 14.67 |
| T100 | - | 2.82 | 35.00 | 12.41 |

^a^ R1-R100 were selected from error-prone PCR plates and T1-T100 were selected from site-specific mutation plates. The colonies were cultivated in LB supplemented with kanamycin at 37°C and 250 rpm for 24 h.

^b^ Colonies with the highest 5-ALA/OD values (highlighted in red) were selected for further study and subsequently redesignated as strains 1 to 20.

**REFERENCES**

Robert, X. and Gouet, P. (2014). Deciphering key features in protein structures with the new ENDscript server. *Nucleic Acids Res.* 42 (W1), W320-W324. doi: 10.1093/nar/gku316
